# Recent Advances in Isatin–Thiazole Hybrids: Synthesis, Structural Design, and Biological Application

**DOI:** 10.1002/cbdv.202501989

**Published:** 2025-08-13

**Authors:** Isadora M. G. Andrade, Edson de O. Lima Filho, Caio F. Valadão, Luana da S. M. Forezi, Fernando de C. da Silva

**Affiliations:** ^1^ Laboratório De Síntese Orgânica Aplicada, Instituto de Química Universidade Federal Fluminense Niterói Rio de Janeiro Brazil

**Keywords:** heterocycles, indole, molecular hybridization, organic synthesis, pharmacophore, small molecules

## Abstract

Isatin–thiazole hybrids are considered privileged chemical scaffolds due to their broad spectrum of pharmacological properties, making them attractive candidates for drug development. As a result, isatin–thiazole derivatives have emerged as a prominent class of hybrid heterocycles and have been the focus of extensive research in recent years, aiming to address gaps in the discovery of potent new drugs. This review presents a comprehensive survey of the synthetic strategies employed to obtain isatin–thiazole derivatives, highlighting the key reactive sites of the isatin core. In addition, it summarizes the biological activities of isatin–thiazole compounds that exhibit promising anticancer, anticonvulsant, anti‐HIV, anti‐inflammatory, antidiabetic, and antioxidant properties. The goal of this review is to provide an updated and thorough overview of the synthesis and biological activities for potential applications of isatin–thiazole derivatives, based on studies published up to 2024.

## Graphical abstract

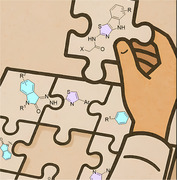

## 1 Introduction

Isatin is a versatile and valuable scaffold in pharmaceutical chemistry, extensively used in the design of novel compounds with enhanced biological activities. Isatin derivatives have exhibited a broad spectrum of pharmacological effects, including anticancer [1, 2], antibacterial [3, 4, 5], antifungal [5], antidiabetic [6], anticonvulsant [7, 8, 9], antitubercular [10], anti‐HIV [11, 12], anti‐viral [11, 13, 14], antioxidant [5, 15, 16], anti‐glycation [17], anti‐malarial [18, 19], anti‐inflammatory [20, 21], and immunosuppressive activity [7], among others. These compounds occur naturally in plants, marine organisms, and human biological fluids [22, 23]. Isatin itself was first synthesized in 1840 by Erdmann and Laurent. It is a stable, bright orange solid that is commercially available on a large scale. Owing to its remarkable biological potential, isatin has been extensively investigated in drug discovery, particularly through molecular hybridization (MH) strategies designed to develop novel therapeutic agents [24, 25, 26, 27, 28, 29, 30].

Thiazole is a widely explored heterocyclic scaffold in medicinal chemistry, present in natural compounds such as thiamine (vitamin B1) and various synthetic drugs [31]. Its stability, structural flexibility, and ability to engage in multiple molecular interactions confer pharmacological properties, including its use in the treatment of allergies [32], hypertension management [33], inflammation reduction, schizophrenia [34], and antibacterial and anti‐HIV activity [35]. Additionally, thiazole derivatives exhibit a broad spectrum of pharmacological activities, including hypnotics [36], analgesics [37], fibrinogen receptor antagonists with antithrombotic activity [38], bacterial DNA gyrase B inhibitors [39], antitumor and cytotoxic activity [40], as well as antifungal and antiviral properties [41].

Given the therapeutic potential of both isatin and thiazole, their combination through MH represents a promising strategy for the development of novel bioactive compounds. This approach involves the covalent conjugation of two or more pharmacophoric units from different bioactive molecules, with or without a spacer. MH not only enables new mechanisms of action but also enhances the biological properties of the original components, resulting in hybrid molecules with optimized therapeutic profiles [42, 43]. Moreover, MH offers several additional advantages, such as overcoming multidrug resistance, minimizing side effects, reducing the risk of drug interactions, and improving the overall safety profile. It also represents a cost‐effective strategy for drug development [42, 43].

Given the complementary bioactive properties associated with these two scaffolds, their MH leads to the creation of a promising class of multifunctional molecules. The synergy between these pharmacophores provides a valuable platform for the development of therapeutic solutions targeting unmet medical needs, particularly in areas, such as cancer, infectious diseases, and inflammation.

In this context, we present a comprehensive review of the biological activities resulting from the MH of isatin and thiazole, along with their structural features and synthetic strategies, with an emphasis on recent advances and their therapeutic potential.

## 2 Isatin

**FIGURE 1:**
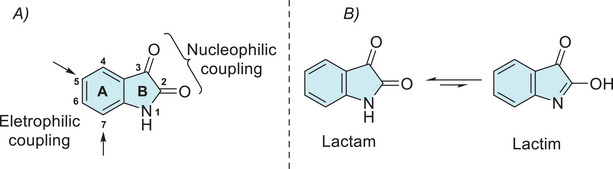
(A) Active sites in the structure of isatins and (B) tautomeric forms of isatin.

The reactivity at the isatin ring A and B sites can be predicted on the basis of electronic density (Figure 1A) [29, 44]. In ring B, nucleophilic sites are located at the nitrogen and oxygen atoms of the C‐2 carbonyl group, whereas the electrophilic sites are contained in the C‐2 and C‐3 carbonyls. In ring A, there is activation at C‐5 and C‐7 sites. The main nucleophilic and electrophilic sites allow a wide range of structural modifications, facilitating their application in the synthesis of new bioactive compounds. The synthetic versatility of isatin arises from its multiple reactive sites, which have been exploited in alkylation, arylation, and acylation reactions. A comprehensive survey of all reactions occurring at its main reactive sites has been conducted. Isatin exhibits a lactam–lactim tautomeric equilibrium, with the lactam form being predominant (Figure 1B) [29, 45]. The existence of both tautomers has been supported by experimental observations, including NMR and IR spectral data [29, 46, 47, 48].

**FIGURE 2:**
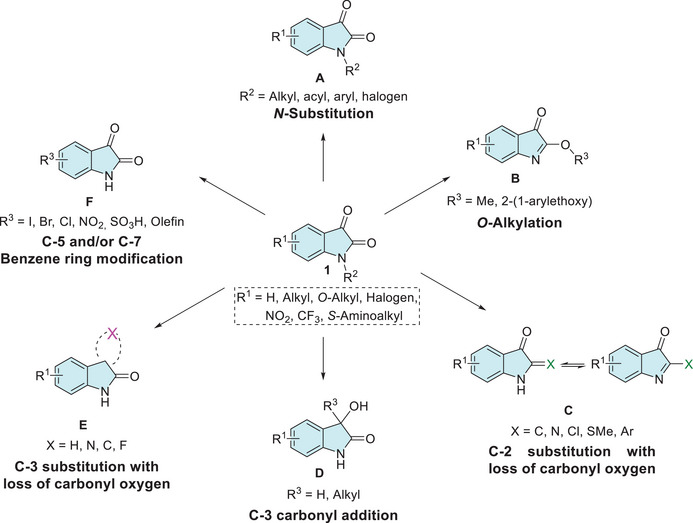
General isatin moiety modification is reported in the reactive sites.

The *N*‐1 site of isatin has a low‐p*K*a value of approximately 10.34, making it easily deprotonated and highly reactive for *N*‐substitution with alkyl, carbohydrate, aryl, acyl, or halogen groups (**A**, Figure 2) [25, 38, 44]. Although isatin is shown to be resistant to *O*‐alkylation, under specific conditions, the lactim form may be favored, leading to the formation of the analog product **B** (Figure 2). Regarding this, only two experimental syntheses have been described: one using silver acetate combined with methyl iodide and another employing boron trifluoride diethyl etherate as a catalyst with trichloroacetimidate (Figure 2) [44, 49, 50]. Modification at the C‐2 position of isatin enables scaffolds, such as dimerization, olefination, halogenation, thioether, arylation, and Schiff's base with hydrazinecarbothioamide or hydrazinecarboxamide (**C**, Figure 2) [51, 52, 53, 54]. The C‐3 carbonyl group of isatin can also undergo various transformations, such as reduction to oxindole, aldol reactions, alkylation via olefins, α‐cross‐coupling, vinylation, alkynylation, hydride anion addition, aldol addition of allenic esters, the Morita–Baylis–Hillman reaction, and trifluoromethylation (**D**, Figure 2) [42, 44, 55, 56, 57, 58, 59, 60, 61, 62, 63, 64] C‐3‐substituted isatins represent an important class of compounds due to their immense biological activities. Many synthetic methods starting from isatin scaffold are described for allowing the loss of carbonyl oxygen to obtain derivatives at C‐3 position, such as imine from hydrazine or hydrazone, Schiff's base, fluorination, ylidene formed by Wittig or by 1,3‐dipolar/inverse reaction, spiro, ketals, reduction for CH_2_, and alkoxyamines hydrochlorides (**E**, Figure 2) [29, 63, 64, 65, 66, 67, 68, 69, 70]. In the case of the benzene ring of isatin, there are just electrophilic substitution reactions at C‐5 and C‐7 positions, whereas no derivatization is reported at C‐4 and C‐6 sites starting from the isatin reactant. For selective C‐5 functionalization methodologies are described for iodination, bromination, chlorination, nitration, sulfonation, and olefination. In C‐5 and C‐7 positions simultaneously, reported just halogenation (–Cl, –Br) and nitration (–NO_2_) (**F**, Figure 2) [71, 72, 73].

## 3 Thiazole

**SCHEME 1:**
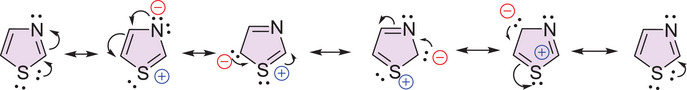
Resonance of the thiazole ring.

Thiazole is a five‐membered aromatic heterocycle, exhibiting high chemical stability and reactivity due to the delocalization of π‐electrons in its conjugated system (Scheme 1) [74, 75, 76, 77]. Among its isomeric forms, 1,3‐thiazole is the most widely studied isomer in pharmacological research. This structure favors intermolecular interactions such as hydrogen bonding, π–π stacking, and coordination with metal ions [74], which are essential for molecular recognition and therapeutic efficacy [75]. Its versatility enables effective interactions with biological targets, particularly through van der Waals forces and intermolecular hydrogen bonds with amino acid residues in receptor proteins [76].

**SCHEME 2:**
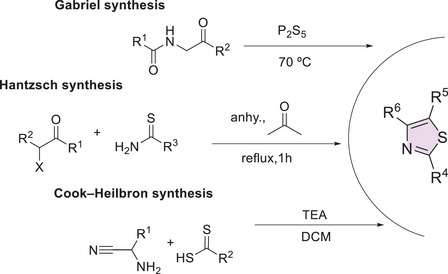
Classical synthetic routes for the construction of the thiazole core.

This structure simultaneously features an electron‐withdrawing group (–C = N–) and an electron‐donating group (–S–). The electronic distribution within the ring, enhanced by the electronegativity of nitrogen and the participation of sulfur's *d* orbitals, contributes to the system's stability and directly influences its reactivity patterns. Electrophilic substitution reactions preferentially occur at the C‐5 and C‐4 positions, whereas nucleophiles tend to attack the C‐2 position [75, 76, 77]. Moreover, the reactivity profile of thiazole can be modulated by the nature of the substituents attached to the ring. Electron‐donating groups increase the basicity and nucleophilicity of the structure, thereby enhancing its potential as a bioactive scaffold in the development of new drugs [75].

Several classical synthetic methods, such as those developed by Hantzsch, Cook–Heilbron, and Gabriel, have been employed in the construction of the thiazole core, using both homogeneous and heterogeneous catalysis (Scheme 2). In addition, modern strategies have enabled the generation of functionalized analogs with high selectivity and yields, further expanding the potential applications of thiazole in drug discovery [75].

## 4 Molecular Hybridization

The pharmaceutical industry has invested heavily in the search for new therapeutic tools to address emerging diseases, as well as in innovations aimed at improving the treatment of well‐established conditions [78, 79]. The implementation of advanced methodologies such as computer‐aided drug design, prediction of physicochemical and structural properties related to drug–receptor interactions (QSAR), automated processes, and novel in vitro and in vivo pharmacological evaluation techniques has proven essential. In this context, the development of new drugs through synthetic strategies such as MH exemplifies the modern approach to compound discovery [43, 78, 79].

**FIGURE 3:**
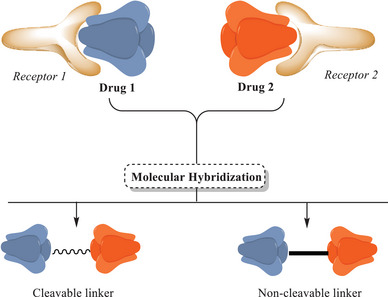
Molecular hybridization model.

The strategy of MH has emerged as a promising tool for drug development. This method involves identifying pharmacophoric subunits within the molecular structures of two or more known bioactive derivatives and generating new hybrid molecular architectures through their appropriate fusion. The development of such compound libraries requires efficient screening methods to obtain meaningful insights into homologous structures, enabling these hybrids to exhibit altered selectivity profiles, dual modes of action, and reduced side effects [78]. A broader interpretation of the concept of MH is based on the Darwinian model of natural and evolutionary selection. In this approach, hybrid daughter molecules are generated through the recombination of structural subunits from parent molecules. However, the current understanding of MH focuses on the method of preparation and the type of pharmacophoric linkage, which may or may not preserve the original functional properties [78, 79, 80]. The strategy of combining molecular units through covalent bonds typically involves the use of a molecular linker, which can be either cleavable or non‐cleavable. A cleavable linker can be considered a prodrug, designed to release the active agent independently upon reaching the target site. In contrast, a non‐cleavable linker can be considered a hybrid drug, leading to a structure capable of maintaining the pharmacophoric properties of its subunits while preserving the hybrid's overall structural integrity throughout the therapeutic process. Furthermore, this type of linkage can impart novel biological activities, resulting in a molecule with unique functional properties [43, 78]. An alternative approach involves the direct integration of pharmacophoric groups via a functional group, forming a new bond that typically occurs in an ester, carbamate, or amide. These compounds are typically susceptible to enzymatic hydrolysis (Figure 3) [81].

An example of the need for hybridization of pharmacophoric units (or drugs) is the treatment of complex heterogeneous diseases. In such cases, administering a single drug is often insufficient to effectively address all manifestations of the disease [82]. Under these conditions, diseases often present multiple pathological aspects that may require different mechanisms of action for effective treatment. Diseases treated with combination therapy pose a health risk due to the lack of information on the action of the drugs in the human body. Antibiotic–antibiotic therapy is a well‐known example, where this strategy aims for synergy between the different drug components to enhance efficacy. However, the lack of comprehensive pharmacokinetic data can cause discrepancies between in vitro results and clinical outcomes, contributing to increased drug resistance, as observed in tuberculosis (TB) treatment [83]. Thus, the integration of multiple pharmacophoric units into a single hybrid compound can provide a broader approach, allowing a single drug to act on different therapeutic targets simultaneously, establishing itself as a monotherapy with a unified pharmacokinetic profile [83]. This strategy can significantly improve disease control by reducing the need for multiple medications and minimizing potential side effects associated with polypharmacy.

Heterocyclic scaffolds are ideal structures for anchoring pharmacophores aimed at producing potential drugs. These compounds enhance physicochemical properties and cellular permeability in vitro, facilitating the oral and biological flexibility of the drug [82, 83].

**SCHEME 3:**
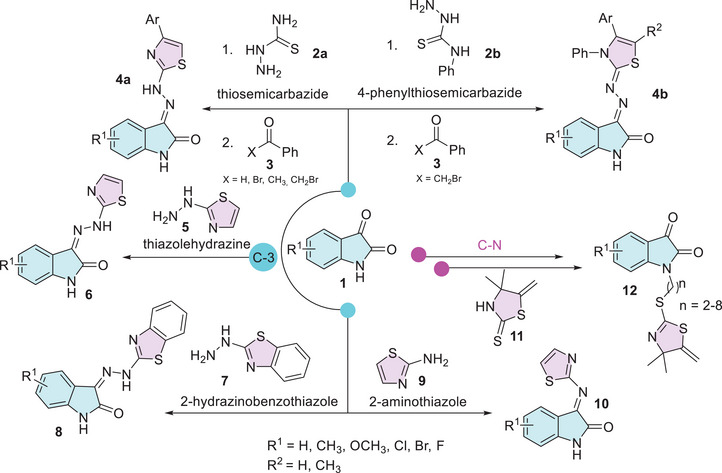
Isatin–thiazole hybrid derivatives using isatin as starting material.

Isatins are versatile heterocycles that have been extensively studied in various contexts, as previously discussed. However, its hybridization with thiazole has received comparatively less attention. Moreover, there are no reports of specific functionalization at ring A. The most common modification involving the thiazole moiety occurs at the C‐3 position, where the loss of oxygen leads to the formation of hydrazine or hydrazone derivatives (Scheme 3). Synthesis methodologies for isatin–linked thiazoles typically target the C‐3 carbonyl group. These include the formation of hydrazones via reactions with thiosemicarbazide **2a** or 4‐phenylthiosemicarbazide **2** in combination with benzaldehyde, benzophenone, or bromobenzyl **3**. This sequence leads to cyclization and the formation of the thiazole nucleus through a one‐pot multicomponent reaction (**4a**,**b**) [84, 85, 86, 87, 88]. The thiazole moiety is commonly employed for functionalization, such as the formation of Schiff's base at the C‐3 carbonyl position through reactions with amino or hydrazine derivatives of thiazole, specifically thiazole hydrazine (**5**), 2‐hydrazinobenzothiazole (**7**), and 2‐aminothiazole (**9**) (Scheme 3) [45, 89, 90, 91, 92]. The nitrogen atom of isatin has been functionalized in a two‐step process using thiazole, with thiazolidine‐2‐thione **11** serving as a key moiety in the final step (Scheme 3) [93].

**FIGURE 4:**
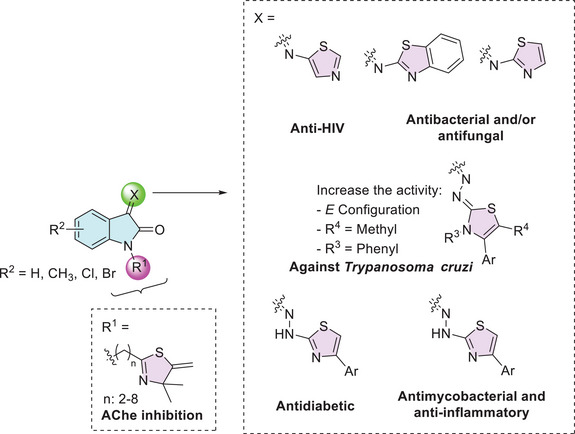
Isatin–thiazole moiety's biological activities.

The isatin–thiazole hybrids are important pharmacophores due to their ability to interact effectively with enzymes, enabling them to combat various diseases. For example, Davis et al. reported the importance of the 2‐thiazoline moiety present at the *N*‐1 site of isatin, along with the free C‐2 and C‐3 carbonyls, in inhibiting the enzyme acetylcholinesterase (AChE), which is associated with diseases such as Parkinson's, Alzheimer's, and bulbar palsy in humans (Figure 4) [93]. At the C‐3 position of isatin, there are more examples containing thiazole moiety. Pawar et al. designed isatin derivatives as anti‐HIV that showed good binding affinity for non‐nucleoside binding pocket (NNBP) site in reverse transcriptase (RT) enzyme [94, 95]. Chohan et al. as well as Sail et al. reported the use of isatin–thiazole as a ligand to afford complexation with metal(II), where these combinations showed affinity as antibacterial and/or antifungal activity [90, 92]. Barros Freitas et al. described the isatin analog as having anti–*Trypanosoma cruzi* activity for trypomastigotes and studied the variation of substituents on thiazole, observing that the *E* configuration, as well as methyl at C‐5 position and phenyl group at *N*‐3, increases the activity [88]. Solangi et al. find that isatin–thiazoles act as potential antidiabetic agents, with binding interactions of the active molecules with α‐amylase and α‐glucosidase in in vitro and in silico studies [84]. Veeranna et al. reported that isatin derivatives exhibited antimycobacterial activity, which is used to treat TB, and good anti‐inflammatory activity against matrix metalloproteinase‐2 (MMP‐2) with inhibition around 80% (Figure 4) [85].

## 5 Isatin–Thiazole Hybrids and Their Biological Applications

Several biological activities have been attributed to isatin–thiazole hybrids, such as anti‐HIV [96], anti‐inflammatory [97], anticancer [98], anticonvulsant [99], antidiabetic [84], antimicrobial [23], antioxidant [23], and cytotoxic activity [23]. These diverse biological effects highlight the therapeutic potential of isatin–thiazole hybrids, making them promising candidates for further investigation. In the following sections, we will delve deeper into the specific mechanisms underlying these activities, as well as recent advancements in the design and synthesis of these hybrid compounds.

### 5.1 Anti‐HIV

HIV‐1 is responsible for most HIV infections worldwide, compared to HIV‐2, the other main type of the virus. Recent reports indicate that by the end of 2022, approximately 39 million people were living with HIV‐1, and 630 000 deaths were attributed to HIV‐1‐related complications. In addition, HIV‐1 can increase susceptibility to various infections, including TB, cryptococcal infection, histoplasmosis, and severe bacterial infections, as well as co‐infections with hepatitis B and C. It is also associated with comorbidities, such as cardiovascular, renal, and hepatic disorders, and certain types of cancer. In the future, managing co‐infections with emerging viruses such as SARS‐CoV‐2 (COVID‐19) and monkeypox will pose additional challenges for clinicians treating people living with HIV‐1 [100].

**SCHEME 4:**
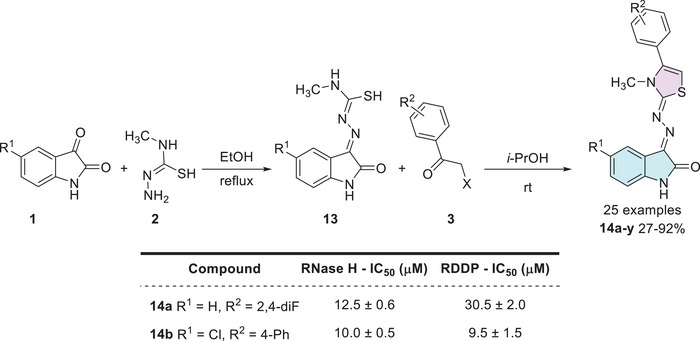
Synthetic route of compounds **14a**–**y** and IC_50_ values for the most active compounds against RNase H and RDDP. RDDP, RNA‐dependent DNA‐polymerase.

HIV‐1 (RT) is an essential enzyme for viral replication, responsible for converting viral RNA into DNA. This process enables the integration of the viral genetic material into the host cell's DNA. Inhibiting this enzyme is a crucial strategy for controlling or slowing the progression of the disease [101]. In the context, Meleddu et al. investigated the anti‐HIV‐1 efficacy of 25 isatin–thiazole compounds, which were synthesized in two steps. First, starting from isatin derivative **1** with a nucleophilic donor, 1‐amino‐3‐methylisothiourea **2**, to form a Schiff's base **13** as an intermediate, which then reacts with 𝛼‐haloacetophenones **3** for thiazole ring formation, affording the desired products, **14a–y** (25 examples), with yields varying from 27% to 92% (Scheme 4). However, there were just two examples, **14a** (R^1^ = H, R^2^ = 2,4‐diF) and **14b** (R^1^ = Cl, R^2^ = 4‐Ph), which showed good results against HIV‐1 (RT) = (i) for ribonuclease H (RNase H), with IC_50_ (µM) values of 12.5 ± 0.6 and 10.0 ± 0.5, respectively; and (ii) RNA‐dependent DNA‐polymerase (RDDP) IC_50_ (µM) values of 30.5 ± 2.0 and 9.5 ± 1.5, respectively. Additionally, they have also studied molecular docking using QMPL default settings, focusing on the most active compounds, that is, **14a** and **14b**, for the RNase H inhibitory activity and polymerase activity, resulting in **14b** being better accommodated compared to **14a** (Scheme 4) [96].

**FIGURE 5:**
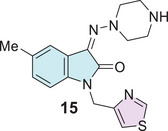
Isatin–thiazole **15** was revealed as an anti‐HIV agent after molecular docking.

Pawar et al. explored a molecular docking study for anti‐HIV also designed isatin derivative drugs, where three compounds were reported with good binding affinity for NNBP of RT enzyme; however, this study contains only one isatin–thiazole **15** type (Figure 5) [94].

### 5.2 Anti‐Inflammatory

Inflammation is a defensive response of living tissue triggered by damage caused by harmful chemicals, physical injury, or bacterial and microbial agents. This response plays a crucial role in combating infections and restoring normal physiological functions, serving as the body's first line of defense [102]. To manage pain, non‐steroidal anti‐inflammatory drugs (NSAIDs) are commonly used. However, long‐term use of NSAIDs is often associated with side effects, including gastrointestinal disturbances, hepatotoxicity, tissue injury, widespread edema, and an increased risk of bleeding [103].

**SCHEME 5:**
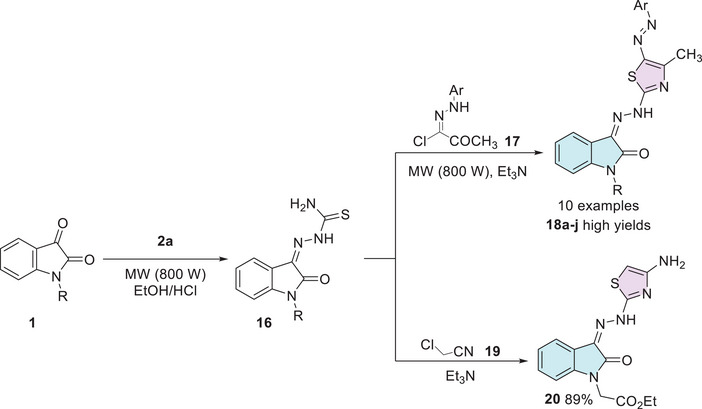
Synthetic route of compounds **18a–j** and **20** and anti‐inflammatory activity.

To explore the anti‐inflammatory activities, Amin et al. synthesized a series of isatin derivatives.[97] Starting from *N*‐1 substituted isatin **1** and using thiosemicarbazide **2a**, a condensation reaction was made, obtaining a thiosemicarbazone **16** as an intermediate, which was used to react with (*E*)‐2‐oxo‐*N*‐arylpropanehydrazonoyl chloride **17** to afford the desired isatin–thiazole hybrids **18a–j** in high yields (**18a**: R = CH_2_CH = CH_2_, Ar = 4‐MePh; **18b**: R = CH_2_CH = CH_2_, Ar = Ph; **18c**: R = COMe, Ar = 4‐MePh). The compound **20** was obtained using thiosemicarbazone **16** and 2‐chloroacetonitrile **19**. Where both products (**18a–j** and **20**) were obtained under cyclization, due to the thiol group at thiosemicarbazide reacting as a nucleophile, promoting the S_N_2 reaction, followed by a second attack by the amino group to cycle, with a carbonyl to afford **18a–j**, and with a nitrile to obtain **20** (Scheme 5).

The synthesized compounds **18b**, **18a**, **18c**, and **20** demonstrated the most significant anti‐inflammatory effects compared to the standard drug indomethacin after 4 h of carrageenan administration in albino rats. These compounds showed a marked reduction in edema, with paw thickness measurements of 3.12 ± 0.02 mm for **18b** and **18c**, 3.15 ± 0.02 mm for **18a**, and 3.10 ± 0.01 mm for **20**, compared to 5.5 ± 0.06 mm observed for Indomethacin. In terms of percentage anti‐inflammatory effect, the compounds exhibited values above 170% (**18a**: 172.75%, **18b** and **18c**: 173.7%, **20**: 174.32%), whereas indomethacin was set as 100%. Furthermore, these compounds significantly reduced serum levels of inflammatory biomarkers: PGE2 decreased from 447.44 ± 16.18 ng/L (positive control) to values among 231.15 ± 3.0 ng/L (**18a**), 241.72 ± 7.22 ng/L (**18b**), 241.52 ± 3.14 ng/L (**18c**), and 236.92 ± 3.26 ng/L (**20**), compared to 324.11 ± 8.42 ng/L for Indomethacin; TNF‐α was reduced from 41.7 ± 1.61 ng/L to 21.25 ± 0.42 ng/L (**18a**), 19.81 ± 0.8 ng/L (**18b**), 20.62 ± 0.71 ng/L (**18c**), and 19.95 ± 0.35 ng/L (**20**), all below the Indomethacin value of 30.81 ± 1.5 ng/L; and IL‐1β decreased from 75.32 ± 3.24 to around 40 ng/L in the active compounds, with **18a** (40.4 ± 1.05 ng/L), **18b** (40.62 ± 1.31 ng/L), **18c** (39.46 ± 1.09 ng/L), and **20** (40.28 ± 1.26 ng/L) being more effective than indomethacin (51.1 ± 2.2 ng/L) (Table 1) [97].

**TABLE 1:** Summary of biological activity results of **18a**, **18b**, **18c**, and **20**.

Compounds	Edema paw thickness (mm)	Anti‐inflammatory effect (%)	PGE2 (ng/L)	TNF‐α (ng/L)	IL1‐β (ng/L)
**18a** (R = CH_2_CH = CH_2_, Ar = 4‐MePh)	3.15 ± 0.02	172.75	231.15 ± 3.09	21.25 ± 0.42	40.4 ± 1.05
**18b** (R = CH_2_CH = CH_2_, Ar = Ph)	3.12 ± 0.02	173.7	241.52 ± 3.14	20.62 ± 0.71	39.46 ± 1.09
**18c** (R = COMe, Ar = 4‐MePh)	3.12 ± 0.02	173.7	241.72 ± 7.22	19.81 ± 0.8	40.62 ± 1.31
**20**	3.1 ± 0.01	174.32	236.92 ± 3.26	19.95 ± 0.35	40.28 ± 1.26
**Control positive**	8.73 ± 0.11	—	447.44 ± 16.18	41.7 ± 1.61	75.32 ± 3.24
**Indomethacin**	5.5 ± 0.06	100	324.11 ± 8.4	30.81 ± 1.5	51.1 ± 2.2

The comparison among the active compounds allows interpretation of how specific structural modifications directly influence anti‐inflammatory activity. Compounds **18a** and **18b**, which share the side chain R = CH_2_CH = CH_2_ and differ only in the aromatic ring (Ar = 4‐MePh in **18a** and Ph in **18b**), exhibit similar activity, suggesting that the methyl group on the ring has a limited effect. Compound **18c**, with R = COMe and Ar = 4‐MePh, also maintained high activity, indicating that both unsaturated chains and ketone groups are well tolerated. In contrast, compound **20** showed the best performance, suggesting that polar and electron‐donating groups significantly enhance anti‐inflammatory activity (**18a–j** and **20**, Table 1).

**SCHEME 6:**
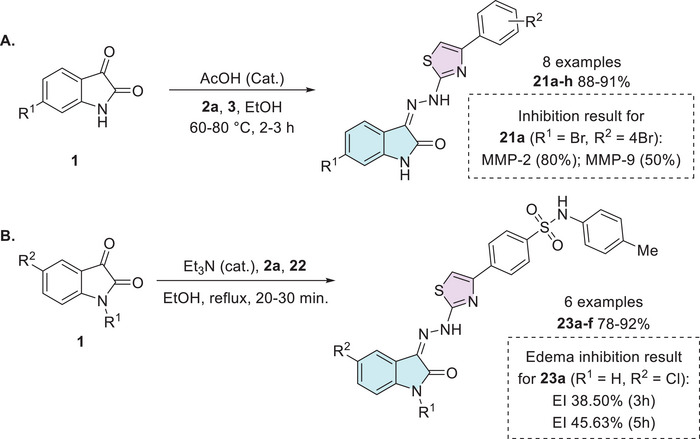
Synthetic route of isatin–thiazole derivatives (A) **21a–h** and (B) **23a–f**, with anti‐inflammatory inhibition for the most active compounds.

Veeranna et al. synthesized eight examples (**21a–h**, 88%–91%) in good to excellent yields of isatin hybrids with thiazole moiety as Schiff's base linked at the C‐3 position of isatin. The synthesis of the conjugates was carried out using isatin and its derivatives **1**, thiosemicarbazide **2a**, and 2‐bromoacetophenone derivatives **3**. Only the compound **21a** (R^1^ = Br, R^2^ = 4‐Br) showed activity as an anti‐inflammatory (Scheme 6) [85]. Cyclooxygenase‐2 (COX‐2) is an enzyme isoform that catalyzes the production of prostaglandins, which are key chemical mediators in the inflammatory response. COX‐2 is specifically upregulated during inflammation, promoting the recruitment of pro‐inflammatory cytokines and chemokines. Selective inhibition of COX‐2 helps to control and reduce inflammation while minimizing the gastrointestinal side effects commonly associated with non‐selective COX inhibition. In this context, the development of novel COX‐2 inhibitors has become a significant focus of pharmaceutical research [104]. Alkorbi et al. reported the synthesis and molecular docking of thiazol‐indolin‐2‐one derivatives **23a–f**, starting from isatin derivative **1**, in the presence of thiosemicarbazide **2a** and 4‐(2‐bromoacetyl)‐*N*‐(*p*‐tolyl)benzenesulfonamide **22**. From six target products (**23a–f**, 78%–92% yields), just one (**23a**: R^1^ = H, R^2^ = Cl) was the most active compound as an anti‐inflammatory agent, exhibited high edema inhibition (EI = 38.50%), and the docking study revealed good fitting into COX‐2 enzyme binding site (Scheme 6) [105].

**SCHEME 7:**
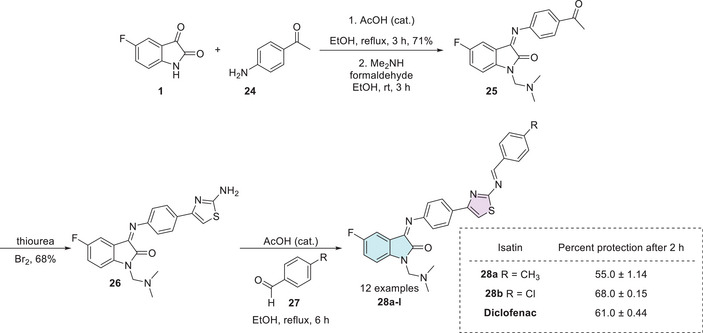
Synthetic route of compounds **28a**–**l** and activity for relevant compounds.

In 2014, Prakash et al. synthesized a series of thiazoles (**28a–l**), 12 examples with no reported yields. The synthesis began with equimolar quantities of 5‐fluoroisatin **1** and *p*‐aminoacetophenone **24** that reacted with aqueous formaldehyde and dimethylamine in ethanol, yielding the intermediate **25**. Subsequently, the compound **25** was treated with thiourea and bromine to form a product containing the thiazole ring **26**. At last, **26** reacts with various substituted benzaldehydes **27** to afford the desired products **28a–l** (Scheme 7). The biological evaluation of the isatin–thiazole hybrids **28a–l** was conducted to assess their potential anti‐inflammatory activity. In an in vitro assay, compounds **28a** (R = CH_3_, 55.0 ± 1.14) and **28b** (R = Cl, 68.0 ± 0.15) demonstrated significant anti‐inflammatory effects, comparable to the reference drug diclofenac (61.0 ± 0.44), as illustrated in Scheme 7. These results suggest that the synthesized compounds possess promising pharmacological potential and warrant further investigation [102, 106].

### 5.3 Anticancer

Cancer is a major cause of mortality and morbidity worldwide, and its incidence is independent of regional human development index (HDI) levels. The number of cancer cases is projected to reach 28.4 million by 2040, representing a 47% increase compared to the 19.3 million cases recorded in 2020. This alarming projection highlights the urgent need for the development of newer, safer, and more effective anticancer agents [107].

**SCHEME 8:**
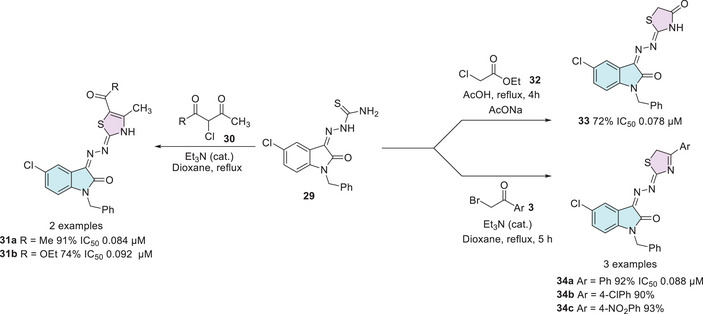
Synthetic methods to achieve compounds **31a**,**b**, **33**, and **34a–c** and IC_50_ values against VEGFR‐2.

Vascular endothelial growth factor receptor‐2 (VEGFR‐2) promotes cell proliferation through the activation of the extracellular signal‐regulated kinase pathway and is a key target in the development of antitumor therapies. Currently, inhibiting the VEGFR‐2 signaling pathway is considered a promising approach in clinical trials for cancer treatment. Vascular endothelial growth factor (VEGF), along with its ligands and receptors (VEGFR), plays a crucial role in the growth and development of new blood vessels. In the neoplastic context, blocking this pathway prevents the recruitment of nutrients and the tumor's blood supply, thereby restricting its growth and progression [108, 109]. Mahmoud et al. reported a series of 2‐indolinone thiazole hybrids (14 molecules) as potential agents for the treatment of renal cell carcinoma as VEGFR‐2 inhibitors based on sunitinib, an FDA‐approved anticancer drug. For the synthesis of the desired compounds, the hydrazone **29**, previously synthesized from *N*‐1 alkylated isatin **1** and **3a**, was used as a common intermediate for the synthesis of **31a,b**, **33**, and **34a–c** (six examples, Scheme 8). The synthesis of **31a,b** pathway occurs from the cyclization of **29** with the dicarbonyl compound **30**. However, the synthesis of **33** occurred between **29** and ethyl chloroacetate **32**. The compounds **34a–c** were synthesized using **29** and α‐bromoacetophenone **3** (Scheme 8). All fourteen compounds were screened and tested against VEGFR‐2. However, just six showed IC_50_ values nearly equipotent to sunitinib (0.075 µM), **31a,b**, **33**, and **34a–c**. Among these, four compounds showed the best results: 0.084 µM (**31a**, R = CH_3_), 0.092 µM (**31b**, R = OEt), 0.078 µM **33**, and 0.088 µM (**34a**, Ar = Ph). These four compounds were also submitted to a docking study, revealing proper fitting into the ATP binding site of VEGFR‐2 [110].

**SCHEME 9:**
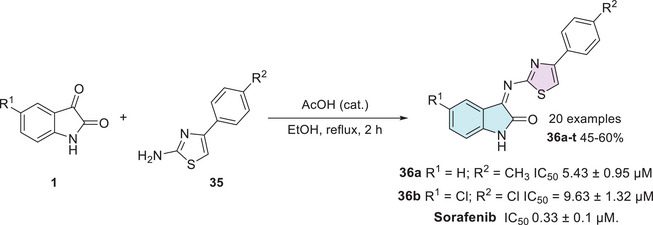
Synthetic route for compounds **36a**–**t** and activity of the best compounds against VEGFR‐2.

Another two recent works published by Shalmali et al. and Al‐Warhi et al. also reported in vitro anticancer screening to inhibit VEGFR‐2. Shalmali et al. synthesized 20 isatin–thiazole derivatives from isatins **1** and 4‐phenylthiazol‐2‐amines **35** through a condensation reaction at C‐3 site to afford **36a–t** in 45%–60% yields, where two of them, **36a** (R^1^ = H; R^2^ = CH_3_) and **36b** (R^1^ = Cl; R^2^ = Cl), inhibited VEGFR‐2 with IC_50_ values of 5.43 ± 0.95 and 9.63 ± 1.32 µM, respectively, in comparison to the standard drug, sorafenib, with an IC_50_ value of 0.33 ± 0.1 µM (Scheme 9). A molecular docking study was made, and the compound **36a** was found to have a maximum binding score, that is, −9.355, and the physicochemical properties were calculated [109].

**SCHEME 10:**
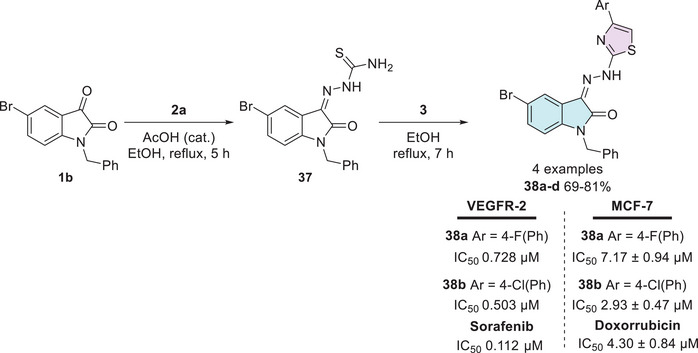
Synthesis and activity of compounds **38a**–**d** against VEGFR‐2 and MCF‐7 cells. VEGFR‐2, vascular endothelial growth factor receptor‐2.

Al‐Warhi et al. [111] synthesized diverse molecules containing the isatin–thiazole scaffold for biological evaluation. The synthetic route to afford the desired products (**38a–d**, 69%–81% yields) started from 5‐bromoisatin **1b**, in three steps involving benzyl bromide for alkylation, followed by reaction with thiosemicarbazide **2a** for condensation to afford **37**, then reaction with 𝛼‐bromoacetophenone **3** as a final step for cyclization to afford **38a–d** (Scheme 10). Among them, compounds **38a** and **38b** exhibited promising dual activity against VEGFR‐2 and MCF‐7 cancer cells. For VEGFR‐2 inhibition, the IC_50_ values were 0.728 µM for **38a** (Ar = 4‐FPh) and 0.503 µM for **38b** (Ar = 4‐ClPh), in comparison to the reference drug sorafenib, which showed an IC_50_ of 0.112 µM. Regarding antiproliferative activity against MCF‐7 cells, the IC_50_ values were 7.17 ± 0.94 µM for **38a** and 2.93 ± 0.47 µM for **38b**, whereas doxorubicin, used as the standard drug, exhibited an IC_50_ of 4.30 ± 0.84 µM. These results highlight compound **38b**, in particular, as a promising dual inhibitor candidate, demonstrating superior antiproliferative activity against MCF‐7 cells compared to doxorubicin and notable VEGFR‐2 inhibition close to that of sorafenib. MCF‐7 cells are a well‐established breast cancer model that expresses estrogen receptor alpha (ERα), a key feature shared by various aggressive breast cancer subtypes. This makes them an essential tool in breast cancer research, particularly given the challenges associated with maintaining ERα expression in cell cultures [111].

**SCHEME 11:**
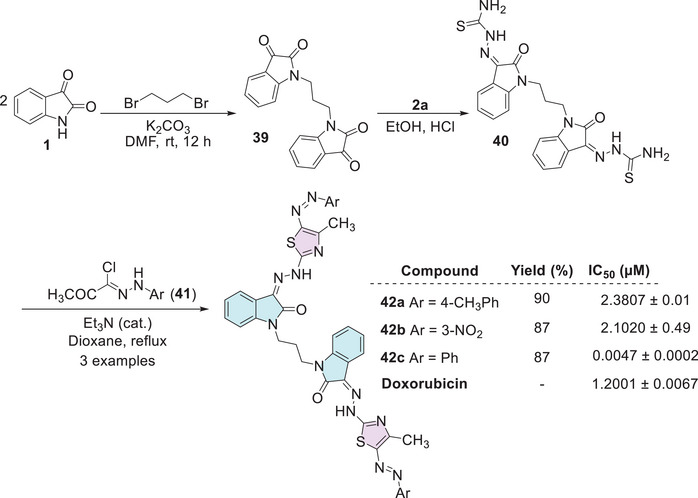
Synthetic route and activity for compounds **42a**–**c** against MCF‐7.

Althagafi et al. developed a series of nine compounds of isatin–thiazole. The synthesis occurs in three steps, starting from isatin **1**, reacting with 1,3‐dibromopropane for the formation of bis‐isatin **39**, followed by the insertion of thiosemicarbazide **2a** at the C‐3 carbonyl position of each isatin structure to afford **40**. Then, the addition of hydrazonoyl chlorides **41** to afford the cyclic final product, that is, the isatin–thiazole derivative (**42a–c**; 87%–90% yields, Scheme 11). The compounds were screened for their cytotoxic activity against the MCF‐7 breast cancer cell line, in comparison to doxorubicin (IC_50_ = 1.2001 ± 0.0067 µM). The three bis‐thiazoles **42a** (Ar = 4‐CH_3_Ph, IC_50_ = 2.3807 ± 0.01 µM), **42b** (Ar = 3‐NO_2_Ph, IC_50_ = 2.1020 µM), and **42c** (Ar = Ph, IC_50_ = 0.0047 ± 0.0002 µM) were evaluated. Notably, compound **42c** demonstrated remarkable potency. Its IC_50_ value was over 250 times lower than that of doxorubicin, highlighting its strong potential as a lead compound for further anticancer drug development [112].

**SCHEME 12:**
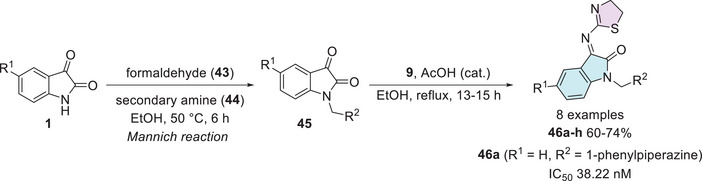
Synthetic route of compounds **46a**–**h** and activity against MCF‐7 for the best compound.

Taher et al. synthesized a series of eight isatin–thiazole derivatives, synthesized in two steps from 5‐substituted isatins **1**, with a methylamination using formaldehyde **43** combined with a secondary amine **44** (piperidine, morpholine, 1‐methylpiperazine, diphenylamine, and 1‐arylpiperazine derivatives) to afford **45**, which reacts with 2‐amino‐4,5‐dihydrothiazole **9** to obtain a condensation product at C‐3 position (**46a–h**) with yields varying from 60% to 74% (Scheme 12). After the products were analyzed for in vitro cytotoxic activity against human breast cancer cells (MCF‐7), using doxorubicin (IC_50_ = 5.46 nM) as the reference drug, only **46a** (R^1^ = H, R^2^ = 1‐phenylpiperazine) showed excellent activity, with an IC_50_ value of 38.22 nM. Although less potent than doxorubicin, compound **46a** still demonstrated strong cytotoxicity in the nanomolar range, indicating its potential as a promising lead for further optimization (Scheme 12) [113].

**SCHEME 13:**
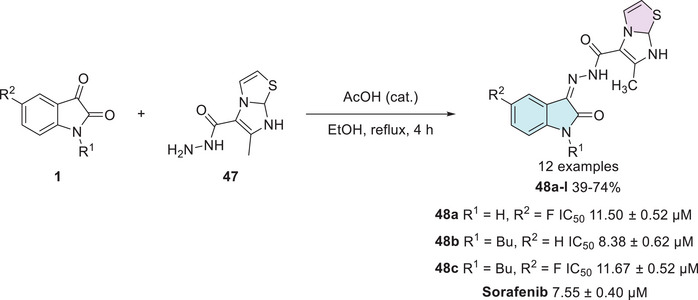
Synthetic route of compounds **48a**–**l** and activity against MCF‐7 for the most active compounds.

Alshaye et al. designed and synthesized an isatin–thiazole scaffold starting from isatin **1** combined with carbohydrazide **47** in a single step to afford the corresponding Schiff's base at C‐3 site with 12 products **48a–l**, with 39%–74% yields (Scheme 13). The compounds were evaluated for their potential against MCF‐7 breast cancer. Only three compounds in the developed series, **48a** (R^1^ = H, R^2^ = F), **48b** (R^1^ = Bu, R^2^ = H), and **48c** (R^1^ = Bu, R^2^ = F), showed good activity, with IC_50_ values of 11.50 ± 0.52, 8.38 ± 0.62, and 11.67 ± 0.52 µM, respectively, when compared to the reference drug sorafenib, which exhibited an IC_50_ of 7.55 ± 0.40 µM. Among them, compound **48b** demonstrated the closest activity to sorafenib, suggesting potential for further structural refinement [114].

**SCHEME 14:**
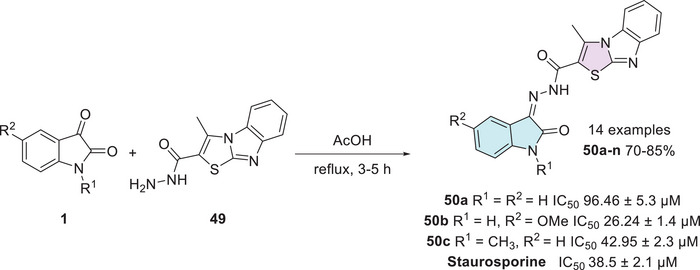
Synthetic route of compounds **50a**–**n** and activity against breast cancer for the most active compounds.

Cyclin‐dependent kinase 2 (CDK2) plays a crucial role in regulating the cell cycle, particularly during the process of cell division, ensuring that it proceeds in a controlled and orderly manner. Dysregulation in the expression or activity of CDK2 is associated with the development of neoplasms, making its inhibition a promising strategy for controlling cancer cell proliferation [115]. In this context, Eldehna et al. reported the synthesis of an isatin derivative featuring a thiazol[3,2‐a]benzimidazole (TBI) motif, linked via a cleavable hydrazide linker, as a potential anticancer CDK2 inhibitor. The synthetic methodology uses isatin derivative **1** with thiazole‐2‐carbohydrazide derivative **49** under condensation reaction at the C‐3 position of isatin to afford **50a–n** (14 examples, 70%–85%, Scheme 14). The hybrids **50a**, **50b**, and **50c** from fourteen examples tested displayed potent dual activity against the examined cell lines and were thus selected for further investigations. Where **50a** (R^1^, R^2^ = H), **50b** (R^1^ = H, R^2^ = OMe), and **50c** (R^1^ = CH_3_, R^2^ = H) showed significant CDK2 inhibitory activity, with IC_50_ values of 96.46 ± 5.3 µM, 26.24 ± 1.4 µM, and 42.95 ± 2.3 µM, respectively, in comparison to the reference drug staurosporine, which exhibited an IC_50_ of 38.5 ± 2.1 µM. Notably, compound **50b** showed superior inhibition compared to staurosporine, suggesting it as a promising lead candidate for CDK2‐targeted therapy [116].

**SCHEME 15:**
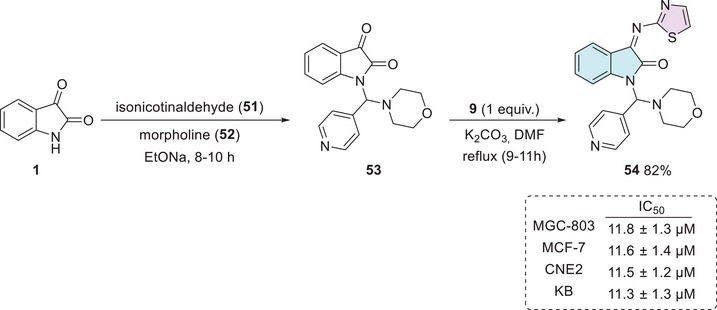
Synthetic route to afford compound **54** and activity against MGC‐803, CNE2, and KB.

MGC‐803 is a cell line derived from gastric adenocarcinoma and is widely used as an experimental model for studying various types of gastric tract cancers [117]. In this context, Abu‐Hashem and Al‐Hussain developed a series of isatin derivatives. The synthetic procedure occurs in two steps starting from isatin **1**, isonicotinaldehyde **51**, and morpholine **52** via one‐pot in the first step to afford **53** as an intermediate, followed by condensation at the C‐3 position using thiazol‐2‐amine **9**, to afford **54** in 82% yield (Scheme 15). The isatin–thiazole compound **54** demonstrated significant activity against human breast adenocarcinoma cells (MCF‐7). Compound **54** demonstrated cytotoxic activity against various human cancer cell lines, including human gastric carcinoma (MGC‐803), breast adenocarcinoma (MCF‐7), nasopharyngeal carcinoma (CNE2), and oral carcinoma (KB) cells. The IC_50_ values obtained were 11.8 ± 1.3 µM (MGC‐803), 11.6 ± 1.4 µM (MCF‐7), 11.5 ± 1.2 µM (CNE2), and 11.3 ± 1.3 µM (KB), which are comparable to those of the reference drug 5‐Fluorouracil, which exhibited IC_50_ values of 10.7 ± 1.2, 10.5 ± 1.1, 10.3 ± 1.3, and 10.1 ± 1.1 µM against the same respective cell lines (Scheme 15) [118]. Notably, KB cells, derived from oral carcinoma, are known for their resistance to various treatments, making them a challenging model for drug development. Research on these cells has gained increasing relevance, as the onset of this type of neoplasia is influenced not only by genetic factors but also by common risk behaviors such as alcohol consumption, smoking, and poor oral hygiene. Given the high toxicity and resistance associated with current treatments, the search for new therapeutic molecules, such as isatin derivatives, is crucial for improving oral cancer treatment [119, 120].

**SCHEME 16:**
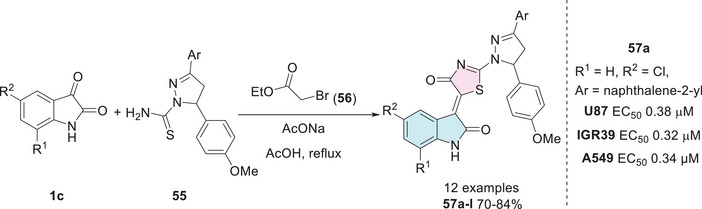
Synthetic route and activity of compounds **57a**–**l** against IGR39, U87, and GR39.

Meleddu et al. synthesized compounds to obtain isatin–thiazole hybrids, beginning with isatins **1c**, which were reacted with **55** and ethyl bromoacetate **56** to afford **57a**–**l** (Scheme 16). Among the tested cell lines, compound **57a** (R^1^ = H, R^2^ = 5‐Cl, Ar = naphthalene‐2‐yl) exhibited notable biological activity and was the most potent within all the tested compounds, with EC_50_ values ranging from 0.01 µM against H1299 to 0.38 µM against U87 cells, and 0.33 µM against IGR39, and 0.34 µM against A549 [121].

IGR39 is a cell line derived from melanoma, a type of cancer that originates in melanocytes, the cells responsible for melanin production. This cell line has been instrumental in advancing research on metastatic melanoma, a highly aggressive and treatment‐resistant form of cancer. The incidence of melanoma has been rising in the population, largely due to increased exposure to ultraviolet (UV) radiation [122, 123].

**SCHEME 17:**
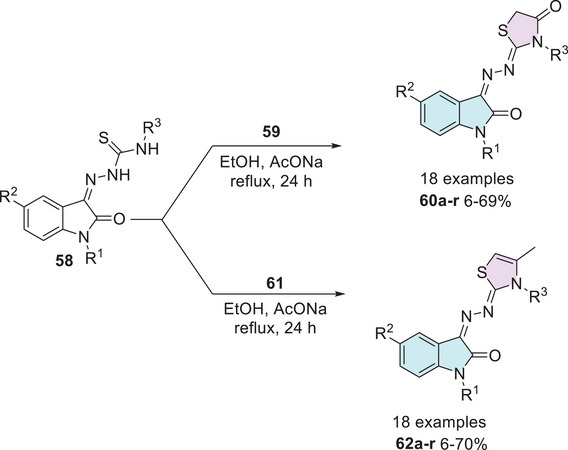
Synthetic route of compounds **60a–r** and **62a–r**.

Yousef et al. [124] in 2020 synthesized isatin–thiazole compounds (**60a–r** and **62a–r**) as anticancer agents. The synthetic route starting from *N*‐1 alkylated or free isatin **1** reacts with thiosemicarbazide derivative **2** to obtain a condensation product **58** as an intermediate that reacts with 2‐chloroacetic acid **59** to afford **60a–r** (6%–69%). To obtain **62a–r**, **61** was used via cyclization (6%–70%). The compounds were obtained as *E*/*Z*‐diastereomers (Scheme 17).

However, just some compounds such as **60a** (R^1^ = H, R^2^ = NO_2_, R^3^ = CH_3_), **60b** (R^1^ = Prop, R^2^ = NO_2_, R^3^ = CH_3_), **62a** (R^1^ = Prop, R^2^ = H, R^3^ = Et), **62b** (R^1^ = Prop, R^2^ = H, R^3^ = CH_3_), **62c** (R^1^ = Prop, R^2^ = CH_3_, R^3^ = Et), **62d** (R^1^ = Prop, R^2^ = CH_3_, R^3^ = CH_3_), and **62e** (R^1^ = H, R^2^ = NO_2_, R^3^ = CH_3_) showed the best activity against (HepG2, liver), (MCF‐7, breast), and (HT‐29, colon) when compared to the reference drug doxorubicin (Table 2). A docking study was performed for **60a,b** and **62a–e**, where **60a** was observed with the highest binding affinity without differences in binding interactions or docking energy between the two diastereoisomers [124].

**TABLE 2:** IC_50_ (µM) values of compounds **60a**,**b** and **62a**–**e** against HepG2, MCF‐7, and HCT‐29.

Compounds	HepG2	MCF‐7	HCT‐29
**60a** (R^1^ = H, R^2^ = NO_2_, R^3^ = CH_3_)	27.59 ± 1.9	8.97 ± 0.7	5.42 ± 0.6
**60b** (R^1^ = Prop, R^2^ = NO_2_, R_3_ = CH_3_)	4.97 ± 0.3	5.33 ± 0.4	3.29 ± 0.2
**62a** (R^1^ = Prop, R^2^ = NO_2_, R^3^ = CH_3_)	9.91 ± 1.0	14.27 ± 1.3	7.71 ± 0.9
**62b** (R^1^ = Prop, R^2^ = H, R^3^ = CH_3_)	9.02 ± 1.0	10.48 ± 1.1	6.24 ± 0.4
**62c** (R^1^ = Prop, R^2^ = CH^3^, R^3^ = Et)	7.38 ± 0.8	9.51 ± 0.9	5.71 ± 0.7
**62d** (R^1^ = Prop, R^2^ = CH_3_, R^3^ = CH_3_)	8.14 ± 0.9	7.81 ± 0.6	4.16 ± 0.2
**62e** (R^1^ = H, R^2^ = NO_2_, R^3^ = CH_3_)	6.85 ± 0.5	7.81 ± 0.6	4.01 ± 0.6
**Doxorubicin**	4.50 ± 0.2	4.17 ± 0.2	4.01 ± 0.4

**SCHEME 18:**
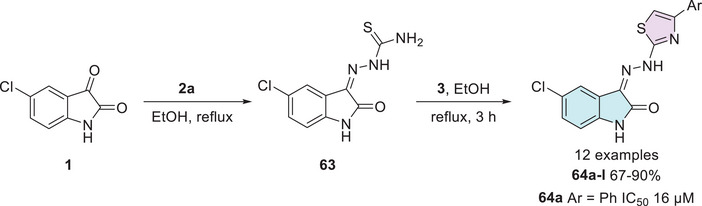
Synthetic route and activity for compounds **64a**–**l** against SCLC. SCLC, small‐cell lung cancer.

Multidrug‐resistant cancer cell lines, such as NCI‐H69AR, play a crucial role in investigating therapeutic strategies for overcoming drug resistance in lung cancer [125, 126]. In the search for new anticancer agents, Eldehna et al. [25] synthesized a series of hydrazonoindolin‐2‐ones (**64a**–**l**), starting from isatin derivatives **1**, which were condensed with thiosemicarbazide **2a** to afford intermediate isatin Schiff base derivatives **63** at the C‐3 position. Then, these intermediates **63** were cyclized with several aryl α‐bromoketones **3** to obtain **64a–l** as the final products (12 examples, 67%–90% yields, Scheme 18).

The compounds were tested, and only **64a** (Ar = Ph) exhibited significant antiproliferative activity against the multidrug‐resistant small‐cell lung cancer (SCLC) cell line NCI‐H69AR, with an IC_50_ value of 16 µM [25]. CLC, including the NCI‐H69AR line, is an aggressive and highly lethal malignancy characterized by high mutational burden, rapid metastasis, and frequent recurrence. Strongly associated with smoking, this cancer type is also notable for its ability to evade immune surveillance by downregulating antigen presentation, allowing tumor cells to remain undetected by the immune system. These features underscore the urgency of developing selective and effective therapeutic agents [127, 128, 129]. In addition to its activity against NCI‐H69AR, the cytotoxicity of compound **64a** was evaluated in three non‐tumorigenic cell lines: IEC‐6 (intestinal), MCF‐10A (mammary epithelial), and Swiss‐3T3 (fibroblast). Notably, compound **64a** demonstrated a mean tumor selectivity index of 1.8, exceeding that of the reference drug Sunitinib (1.4), thereby reinforcing its potential as a promising anticancer agent with selective cytotoxic properties. Furthermore, although not extensively discussed in this study, **64a** was shown to reduce phosphorylated Rb protein levels and increase G1 phase cell population, suggesting that its antiproliferative effect may be mediated through inhibition of cyclin‐dependent kinases and interference in cell cycle progression (Scheme 18) [25].

### 5.4 Anticonvulsant

Epilepsy is a chronic neurological disorder characterized by recurrent, unprovoked seizures, which are typically classified as either partial (focal) or generalized. Approximately 50 million people worldwide are affected by epilepsy, with an estimated 5 million new cases diagnosed each year. Seizures often present with transient symptoms such as altered consciousness or awareness, along with impairments in motor function, sensory perception (including hearing, vision, and taste), mood regulation, and other cognitive processes. In addition, individuals with epilepsy frequently experience comorbid conditions such as anxiety and depression. Notably, around 30% of patients are resistant to conventional antiepileptic therapies. Consequently, the development of novel antiepileptic drugs remains a critical and active area of research [130].

**SCHEME 19:**
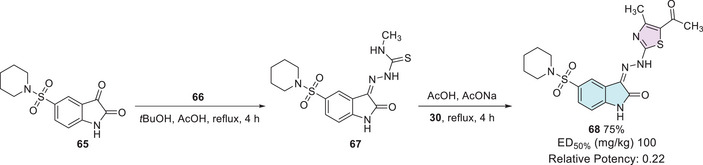
Synthetic route and activity of compound **68**.

Fayed et al. synthesized several compounds using three different synthetic routes. To afford the desired isatin–thiazole **68**, the synthesis started from the 5‐sulfonyl‐substituted isatin precursor **65**, which reacted with a thiosemicarbazide derivative **66** to form intermediate **67**. This intermediate **67** was then reacted with 2‐chloro‐1,3‐diketone **30** to yield the final cyclic product **68** with 75% yield (Scheme 19). After investigation, it was found that isatin–thiazole derivatives have in vivo anticonvulsant activity, providing 50% protection at a dose as low as 100 mg/kg (relative potency of 0.22). Histopathological analysis of the liver revealed mild‐to‐moderate alterations, with compound **68** displaying more evident apoptotic changes. All compounds caused only discrete renal histological alterations. Finally, an in silico prediction supported their drug‐likeness, indicating good oral bioavailability, absence of mutagenicity (AMES test), low probability of severe toxicity, and favorable synthetic accessibility [99].

**SCHEME 20:**
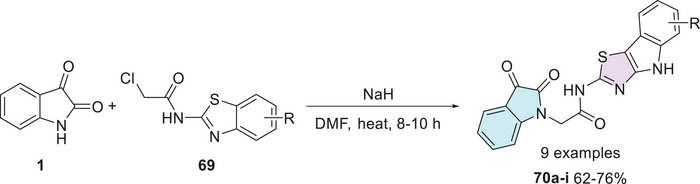
Synthetic route for compounds **70a**–**i**.

Gamma‐aminobutyric acid (GABA) is the primary inhibitory neurotransmitter in the central nervous system (CNS), with the GABA receptor playing a key role in modulating seizure activity. By inducing neuronal hyperpolarization, this receptor exerts an anticonvulsant effect, making it a crucial target for epilepsy treatment [131]. In search of novel anticonvulsant agents, Nath et al. synthesized nine isatin–thiazole derivatives (**70a–i**, 62%–76% yields) starting from **1** via *N*‐1 alkylation, using **69** (Scheme 20). Among the tested compounds, **70a** (R = 4‐Cl) demonstrated the highest activity in preliminary anticonvulsant screening, which included the maximal electroshock seizure (MES) and subcutaneous pentylenetetrazol (scPTZ) models. Furthermore, molecular docking studies revealed that compound **70a** exhibited favorable interactions with GABA receptor binding sites, providing additional support for its potential as a promising anticonvulsant agent (Scheme 20) [132, 133].

### 5.5 Antidiabetic

Diabetes mellitus (DM) is a metabolic and degenerative disorder marked by hyperglycemia. According to the International Diabetes Federation (IDF), around 537 million adults are currently living with diabetes, and the disease was responsible for 6.7 million deaths in 2021. Projections suggest that diabetes cases could rise to 643 million by 2030 and 783 million by 2045 [134].

**SCHEME 21:**
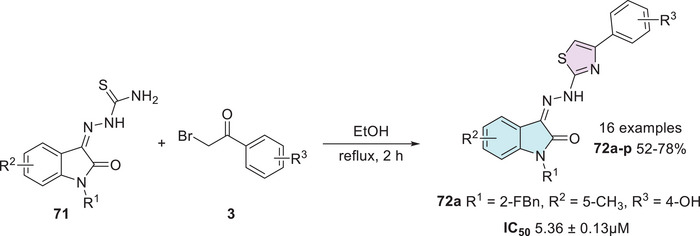
Synthesis of compounds **72a**–**p** for α‐glucosidase inhibition.

The synthesis and evaluation conducted by Xie et al. [135] involved the preparation of isatin–thiazole hybrids. The synthesis was carried out in a single step, starting from substituted isatin bearing a thiosemicarbazide moiety at the C‐3 position as a Schiff base **71** and 𝛼‐bromoacetophenones **3** to afford **72a–p** with 52%–78% yields (Scheme 21). The synthesized compounds were tested against α‐glucosidase, and only **72a** (R^1^ = 2‐FBn, R^2^ = 5‐CH_3_, R_3_ = 4‐OH) exhibited good in vitro activity, with an IC_50_ value of 5.36 ± 0.13 µM, in comparison to the standard drug acarbose, which showed significantly lower potency with an IC_50_ of 817.38 ± 6.27 µM [135, 136].

Alpha‐amylase is an enzyme responsible for breaking down polysaccharides into smaller molecules, such as maltose and dextrose. At the same time, alpha‐glucosidase plays a crucial role in carbohydrate digestion by converting these molecules into absorbable forms like glucose. Inhibiting these enzymes disrupts carbohydrate metabolism, resulting in lower glucose levels in the body, an approach particularly relevant for the management of Type 2 diabetes [137, 138].

**SCHEME 22:**
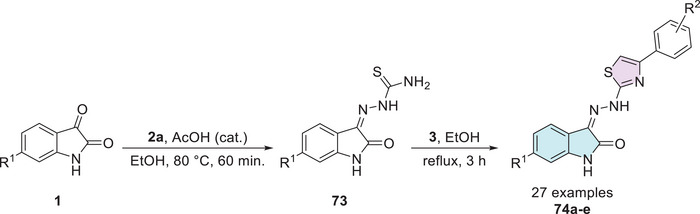
Synthetic route of compounds **74a**–**e**.

In the search for novel antidiabetic agents, Solangi et al. synthesized a series of isatin–thiazole derivatives (27 examples) in two steps starting from substituted isatin **1** and thiosemicarbazide **2a** via condensation in C‐3 carbonyl position in the first step, affording **73** as an intermediate, followed by reaction with 𝛼‐bromoacetophenones **3** to promote the corresponding cyclization product with sulfur and amino groups of Schiff's bases **74a–e** (Scheme 22). A screening of 20 compounds was conducted to evaluate their inhibitory activity against α‐amylase and α‐glucosidase [84].

Among them, five molecules (**74a**: R^1^ = H, R^2^ = 4‐Cl; **74b**: R^1^ = 6‐Br, R^2^ = 4‐Cl; **74c**: R^1^ = H, R^2^ = 4‐Br; **74d**: R^1^ = 6‐Br, R^2^ = 4‐Br; **74e**: R^1^ = H, R^2^ = 3‐NO_2_) exhibited significant enzyme inhibition, highlighting their potential as promising candidates for glucose regulation and diabetes management. The IC_50_ values for α‐amylase ranged from 22.22 ± 0.02 to 27.01 ± 0.06 µM, whereas for α‐glucosidase, they ranged from 24.01 ± 0.12 to 27.11 ± 0.14 µM. In both cases, the compounds demonstrated greater inhibitory activity when compared to the standard drug acarbose (Table 3) [84].

**TABLE 3:** IC_50_ values of compounds **74a**–**e** toward α‐amylase and α‐glucosidase.

Compounds	α‐Amylase (µM)	α‐Glucosidase (µM)
**74a** (R^1^ = H, R^2^ = 4‐Cl)	22.22 ± 0.02	24.01 ± 0.12
**74b** (R^1^ = 6‐Br, R^2^ = 4‐Cl)	26.60 ± 0.06	27.76 ± 0.17
**74c** (R^1^ = H, R^2^ = 4‐Br)	24.74 ± 0.02	26.61 ± 0.11
**74d** (R^1^ = 6‐Br, R^2^ = 4‐Br)	27.60 ± 0.06	20.76 ± 0.17
**74e** (R^1^ = H, R^2^ = 3‐NO_2_)	27.01 ± 0.06	27.11 ± 0.14
**Acarbose**	16.07 ± 0.06	16.66 ± 0.07

**SCHEME 23:**
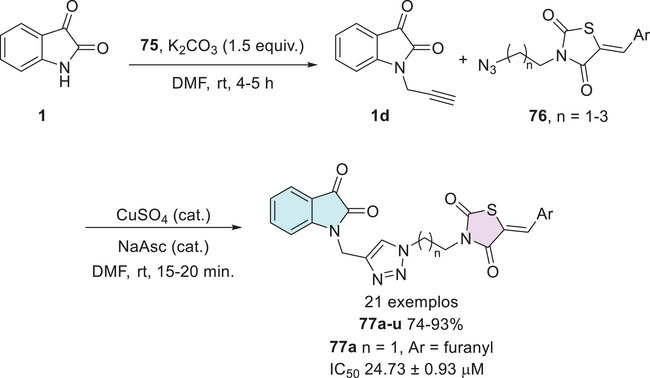
Synthetic route of compounds **77a**–**u** for α‐glucosidase inhibition.

Kaur et al. investigated a series of isatin–thiazole derivatives as potential inhibitors of α‐glucosidase. The synthetic strategy was carried out in two steps. Initially, free isatin **1** was alkylated at the *N*‐1 position using propargyl bromide **75** to afford the corresponding alkyne intermediate **1d**, which subsequently reacts with a thiazole‐containing azide **76** via Huisgen cycloaddition, affording the target isatin–triazole‐thiazole hybrids **77a**–**u** (Scheme 23). Following biological evaluation, including cytotoxicity assessment, molecular docking, binding free energy calculations, and molecular dynamics simulations, compound **77a** (*n* = 1, Ar = furanyl) emerged as the most promising candidate, exhibiting an IC_50_ value of 24.73 ± 0.93 in comparison to the standard drug acarbose, which showed a significantly higher IC_50_ value of 478.07 ± 1.53 µM [125].

**SCHEME 24:**
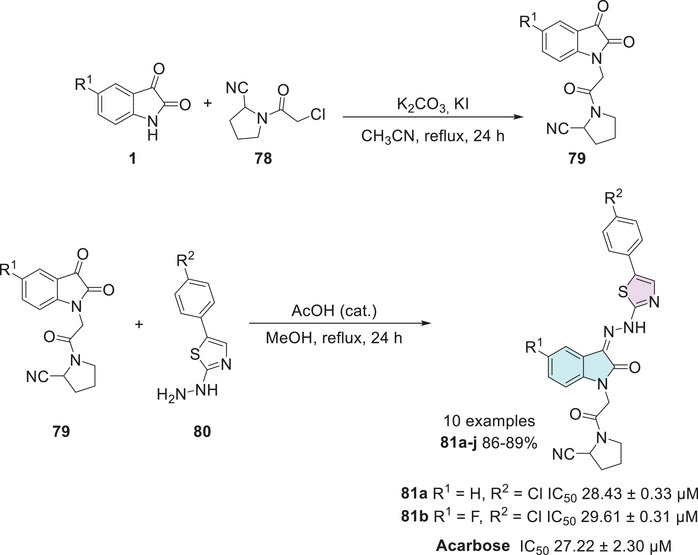
Synthetic route to afford **81a**–**j** and IC_50_ values of the most active compounds.

Patil et al. developed a synthetic route to afford isatin–thiazole derivatives (10 examples). The synthesis was carried out in two steps, initially through *N*‐1 alkylation of substituted isatin **1** using α‐chlorocarbonyl compound **78**, which afforded **79**, followed by condensation at the C‐3 position with thiazole‐based hydrazides **80**, resulting in the target compounds **81a–j** (Scheme 24). Among these structures, only compounds **81a** (R^1^ = H, R^2^ = Cl) and **81b** (R^1^ = F, R^2^ = Cl) demonstrated notable α‐glucosidase inhibitory activity, with IC_50_ values of 28.43 ± 0.33 and 29.61 ± 0.31 µM, respectively, in comparison to the standard drug acarbose (IC_50_ 27.22 ± 2.30 µM), exhibiting a similar activity profile [134].

### 5.6 Antimicrobial

Antimicrobial resistance (AMR) represents an increasingly critical challenge to global public health, as it undermines the effectiveness of infection prevention and treatment across a growing range of pathogens, including viruses, bacteria, fungi, and parasites. This phenomenon has been extensively studied, covering topics from the evolution of resistance and control policies to the discovery of novel antimicrobial agents. Ongoing research and the implementation of effective strategies are essential to limiting the spread of resistant microorganisms and preserving medical advances in the treatment of infectious diseases [139, 140].

**SCHEME 25:**
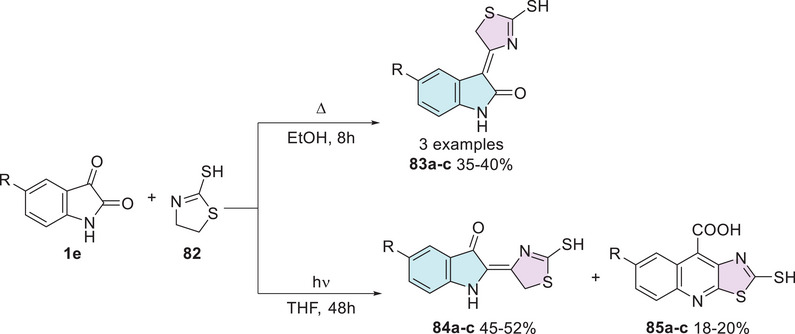
Synthetic route of compounds **83a**–**c**, **84a**–**c**, and **85a**–**c**.

Many years ago, this phenomenon was already a subject of investigation. In 1998, Pardasani et al. [141] described the synthesis of the isatin–thiazole hybrids. The reaction between indol derivatives **1e** (R = H, Br, NO_2_) and thiazoline **82** proceeded via two pathways. The first, for condensation of the carbonyl group at C‐3 position, was carried out under reflux for 8 h, leading to the formation of 3,4′‐dihydro‐3‐[2′‐mercaptothiazolidine]indol‐2‐one (**83a–c**) with 35%–40% yields (Scheme 25). The second pathway for condensation of the carbonyl group at C‐2 position of isatin **1e** with **82** was conducted under photochemical irradiation using a medium‐pressure mercury lamp (∼298–310 nm) for 48 h, leading to the formation of the isomer 2,4‐dihydro‐2‐[2′‐mercaptothiazolidine]indol‐3‐one (**84a–c**) as the major product (45%–52%) and 2‐mercaptothiazolo[5,4‐*b*]quinoline‐4‐carboxylic acid (**85a–c**) as a minor product (18%–20%).

The synthesized compounds were evaluated for their antimicrobial activity against the bacterial strains *Escherichia coli* and *Streptococcus faecalis*, showing inhibition zones for **83a–c** (8.8–12.4, 9.5–10.6 mm, respectively), **84a–c** (8.9–13.4, 9.4–11.6 mm, respectively), and **85a–c** (10.0–11.5, 9.1–10.5 mm, respectively). Additionally, antifungal activity was assessed against *Rhizoctonia solani*, *Fusarium oxysporum*, and *Fusarium solani*, with inhibition zones for **83a–c** (10.0–12.6, 8.5–14.2, 7.8–13.2 mm), **84a–c** (10.2–12.6, 8.4–13.0, 7.9–12.2 mm), and **85a–c** (9.8–11.4, 12.2–12.5, 11.0–13.5 mm), respectively (Table 4). The results indicated that the compounds exhibited moderate antimicrobial activity compared to the reference standards streptomycin and mycostatin [141].

**TABLE 4:** Inhibition zone diameters (mm) of compounds **83a**–**c**, **84a**–**c**, and **85a**–**c**.

Compounds	Escherichia coli	Streptococcus faecalis	Rhizoctonia solani	Fusarium oxysporum	Fusarium solani
**83a** (R = H)	8.8	9.5	10.0	8.5	7.8
**83b** (R = Br)	11.2	9.8	12.2	9.8	10.8
**83c** (R = NO_2_)	12.4	10.6	12.6	14.0	13.2
**84a** (R = H)	8.9	9.4	10.2	8.4	7.9
**84b** (R = Br)	11.2	9.8	12.6	9.8	10.6
**84c** (R = NO_2_)	13.4	11.6	12.6	13.0	12.2
**85a** (R = H)	10.0	9.1	9.8	12.2	11.0
**85b** (R = Br)	10.5	9.8	10.0	12.0	12.6
**85c** (R = NO_2_	11.5	10.5	11.4	12.5	13.5

**SCHEME 26:**
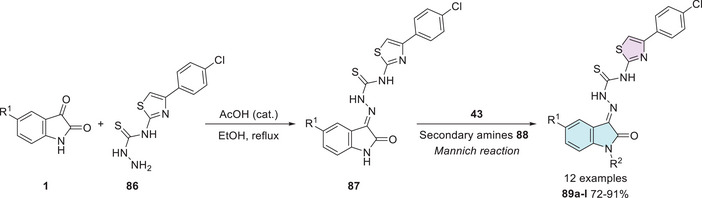
Synthetic route of compounds **89a**–**l**.

Pandeya et al. [12] developed a synthetic method for isatin–thiazole hybrids. The products **88a–l** were synthesized in two steps, using isatin derivatives **1** and a thiosemicarbazide derivative **86** in the first step, resulting in Schiff's bases at C‐3 carbonyl of isatin **87**, whereby *N*‐1 alkylation under the Mannich reaction using formaldehyde (**43**) and secondary amines **88** afforded the desired products **89a–l** with 72%–94% yields (Scheme 26).

These compounds were subsequently investigated as potential antibacterial and antifungal agents, using sulfamethoxazole, trimethoprim, and clotrimazole as positive controls. All compounds exhibited greater activity than sulfamethoxazole, except against *Pseudomonas aeruginosa*. Brominated derivatives demonstrated higher efficacy compared to those containing chlorine substituents or compounds without any derivatization. A similar trend was observed for antifungal activity, with compound **89a** (R^1^ = Br, R^2^ = CH_2_N(CH_3_)_2_) demonstrating the most significant antimicrobial effect against all studied strains. For **89a**, this was reflected in the MIC values, which ranged from 1.2 µg/mL for *Microsporum gypsum* to 78.1 µg/mL for *Candida albicans*. Intermediate values were observed for *Cryptococcus neoformans*, *Microsporum audouinii*, *Trichophyton mentagrophytes*, and *Epidermophyton floccosum* (2.4 µg/mL), followed by *Aspergillus niger* (9.8 µg/mL) and *Histoplasma capsulatum* (19.5 µg/mL) when compared to the standard drug clotrimazole (Table 5) [12].

**TABLE 5:** Summary of the MIC values of compound **89a**.

Microorganism	89a R^1^ = Br, R^2^ = CH_2_N(CH_3_)_2_ (µg/mL)	Clotrimazole
*Cryptococcus neoformans*	2.4	2.4
*Microsporum audouinii*	2.4	4.9
*Trichophyton mentagrophytes*	2.4	2.4
*Epidermophyton floccosum*	2.4	2.4
*Microsporum gypsum*	1.2	2.4
*Histoplasma capsulatum*	19.5	19.5
*Candida albicans*	78.1	0.3
*Aspergillus niger*	9.8	2.4

**SCHEME 27:**
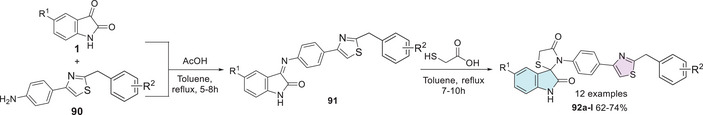
Synthetic route of compounds **92a**–**l**.

In search of new functionalization and more active compounds, in 2010, Mhaske et al. [142] reported the synthesis, characterization, and antimicrobial activity of a series of 11 compounds derived from spiro[indoline‐3,2′‐thiazolidine]‐2,4′‐dione containing a substituted phenyl–thiazole group. The synthesis was carried out initially by reacting isatin **1** with 4‐(2‐arylthiazol‐4‐yl)aniline **90** at the first step for the synthesis of Schiff's bases **91** at C‐3 site of isatin, with the last step involving the addition of thioglycolic acid to afford the spiro compound as the desired product (**92a–l**) with yields ranging from 62% to 74% (Scheme 27).

The derivatives were also analyzed for their in vitro antibacterial activity against Gram‐positive bacteria, where just compounds **92a** (R^1^ = Cl, R^2^ = 4‐Br), **92b** (R^1^ = H, R^2^ = 4‐OCH_3_), and **92c** (R^1^ = Cl, R^2^ = 4‐OCH_3_) were active. For *Bacillus subtilis*, the results of MIC were obtained for the respective compounds **92a** (MIC 70 µg/mL), **92b** (MIC 100 µg/mL), and **92c** (MIC 100 µg/mL). Furthermore, the compounds were active against *Staphylococcus aureus* except for compound **92a**, whereas spiro derivatives **92b** (MIC 80 µg/mL) and **92c** (MIC 90 µg/mL) were active against all tested species. However, regarding antifungal activity, most compounds were inactive, except for compound **92d** (R^1^ = H, R^2^ = H), which demonstrated moderate activity (Table 6). The antifungal potential of compound **92d** was confirmed by its significant inhibition zones against *C. albicans* and *A. niger* when compared to the antifungal standard nystatin. Ciprofloxacin was used as the antibacterial standard, whereas nystatin served as the antifungal reference [142].

**TABLE 6:** Summary of the MIC values and inhibition zone diameters (mm) of **92a**–**d**.

Antibacterial activity MIC (µg/mL)				
	92a (R^1^ = Cl, R^2^ = Br)	92b (R^1^ = H, R^2^ = OCH_3_)	92c (R^1^ = Cl, R^2^ = OCH_3_)	Ciprofloxacin
*Bacillus subtilis*	70	100	100	4
*Staphylococcus aureus*	—	80	90	4
*Escherichia coli*	80	80	90	4

Zone inhibition (mm)		
	92d (R^1^ = H, R^2^ = H)	Nystatin
*Candida albicans*	10.12	20.5
*Aspergillus niger*	9.80	22.1

**SCHEME 28:**
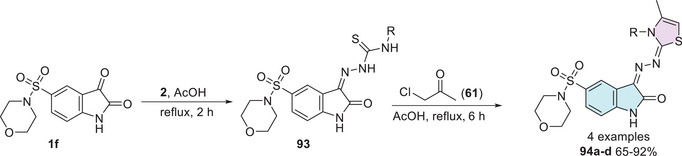
Synthetic route of compounds **94a**–**d**.

The functionalization performed by Farag [143] in 2014 led to the development of four new derivatives of 5‐(morpholinosulfonyl)isatin coupled with thiazole. The synthesis strategy involved the use of 5‐(morpholinosulfonyl)indoline‐2,3‐dione **1f** as the starting material, reacting with thiosemicarbazide derivatives **2** to promote the corresponding condensation product at C‐3 position **93**, which then reacts with 1‐chloropropan‐2‐one **61** to obtain the cyclic isatin–thiazole products **94a–d** with yields varying between 65% and 92% (Scheme 28).

The compounds exhibited notable antibacterial and antifungal activities. Among them, compound **94a** (R = Ph) demonstrated the highest antibacterial potency, with MIC values ranging from 0.06 to 0.49 µg/mL, whereas compound **94b** (R = H) exhibited moderate activity, with MIC values between 0.49 and 3.9 µg/mL against Gram‐positive and Gram‐negative bacteria. In terms of antifungal activity, compound **94a** showed significant inhibition across all tested fungal strains, including *Aspergillus fumigatus*, *Aspergillus clavatus*, *C. albicans*, and *Geotrichum candidum*, with MIC values ranging from 0.06 to 7.8 µg/mL. Conversely, compound **94b** displayed moderate‐to‐good antifungal activity, with MIC values between 0.49 and 7.81 µg/mL, except against *C. albicans*, where no significant effect was observed (Table 7) [143].

**TABLE 7:** Summary of the MIC values of compounds **94a** and **94b**.

MIC (µg/mL)				
	94a (R = Ph)	94b (R = H)	Ampicillin	Amphotericin B
*Staphylococcus aureus*	0.06	0.49	0.06	—
*Staphylococcus epidermidis*	0.49	1.95	0.48	—
*Bacillus subtilis*	0.24	3.9	0.007	—
*Proteus vulgaris*	3.9	31.25	1.95	—
*Klebsiella pneumonia*	1.95	15.63	0.24	—
*Shigella flexneri*	0.98	7.81	0.48	—
*Aspergillus fumigatus*	3.9	15.63	—	0.97
*Aspergillus clavatus*	7.8	7.81	—	1.95
*Geotrichum candidum*	0.98	1.95	—	0.4

**SCHEME 29:**
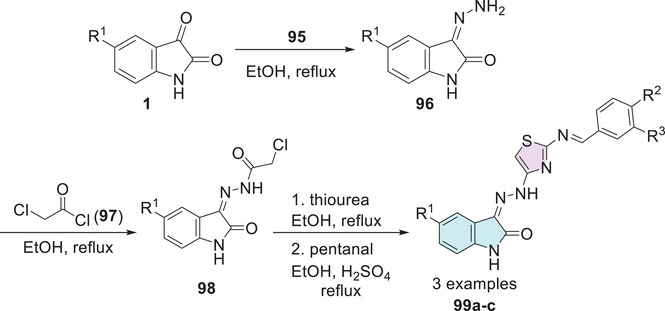
Synthetic route of compounds **99a**–**c**.

Eggadi et al. [23] investigated the antimicrobial and antibacterial activity of isatin‐3‐[*N*2‐(2‐benzalamino‐thiazol‐4‐yl)] hydrazone conjugates. The synthesis of the compounds was carried out in four steps. Initially, isatin derivatives **1** were subjected to a reaction with hydrazine hydrate **95**, resulting in the formation of the corresponding hydrazones **96**. Subsequently, the isatin hydrazones **96** were treated with chloroacetyl chloride **97**, which promotes the alkylation to obtain the chloroacetylated derivatives **98**. In the final step, pentanal reacts with thiourea, leading to the formation of cyclic products, that is, isatin–thiazole products **99a–c** (Scheme 29). In the antibacterial activity profile, compounds **99a** (R^1^ = CH_3_, R^2^ = Cl, R^3^ = H) and **99b** (R^1^ = Cl, R^2^ = Cl, R^3^ = H) (Scheme 29) stood out for their superior efficacy.

Both demonstrated significant activity against the two Gram‐positive bacteria (*B. subtilis* and *S. aureus*), with **99a** exhibiting MIC values of 20 and 16 mm, respectively, and **99b** showing MIC values of 14 and 12 mm. Additionally, both compounds displayed notable activity against the Gram‐negative bacterium *E. coli*, with **99a** achieving 18 mm and **99b** 15 mm. Regarding antifungal activity, the conjugates generally exhibited mild‐to‐moderate activity. Compound **99c** (R^1^ = NO_2_, R^2^ = OH, R^3^ = OMe) stood out by showing the largest inhibition zones against *A. niger* and *Cladosporium verruculosa*, with diameters of 5 and 9 mm, respectively, compared to the standard drug clotrimazole, which presented inhibition zones of 22 and 19 mm, respectively (Table 8). The introduction of electron‐withdrawing groups at Position 5 significantly enhanced the activity of isatin. These groups at Position 5 of isatin and Position 3 of the phenolic ring facilitate transport across the cell membrane, thereby enhancing antimicrobial activity. In vitro assays confirmed that compounds with electron‐donating groups (**99a**) and electron‐withdrawing groups (**99b,c**) at Position 5, along with chlorine substitutions on the aromatic ring, exhibited significant antimicrobial activity. Overall, all compounds demonstrated antibacterial and antifungal activity [23].

**TABLE 8:** Zone of inhibition in mm of compounds **99a**–**c**.

Zone of inhibition (mm)			
	99a (R^1^ = CH_3_, R^2^ = Cl, R^3^ = H)	99b (R^1^ = Cl, R^2^ = Cl, R^3^ = H)	99c (R^1^ = NO_2_, R^2^ = OH, R^3^ = OMe)
*Bacillus subtilis*	20	14	—
*Staphylococcus aureus*	16	12	—
*Escherichia coli*	18	15	—
*Aspergillus niger*	—	—	5
*Cladosporium verruculosa*	—	—	9

**SCHEME 30:**
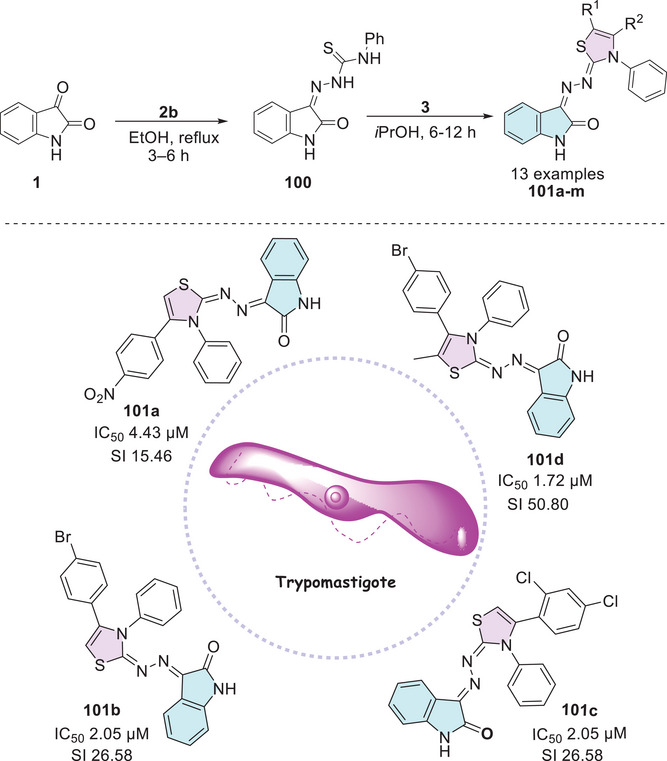
Synthetic route for compounds **101a**–**m** and activity against trypomastigote forms of *Trypanosoma cruzi* for the best compounds.

In 2021, Barros Freitas et al. [88] synthesized several isatin hybrids containing a thiazole nucleus as Schiff's base at C‐3 position, where the route starts from isatin **1** that reacts with 4‐phenylthiosemicarbazide **2b** to obtain (*Z*)‐2‐(2‐oxoindolin‐3‐ylidene)‐*N*‐phenylhydrazine‐1‐carbothioamide **100** as an intermediate, followed by reaction with alpha‐halo ketones **3** to afford the isatin–thiazole conjugates **101a–m** (Scheme 30). The in vitro evaluation of thiazolyl–isatin derivatives **101a–m** against trypomastigote forms of *T. cruzi* (cepa Y) strain generally revealed greater selectivity than the reference drug benznidazole (5.65 µM). Notably, compounds **101a** (IC_50_ = 4.43 µM; R^1^ = H, R^2^ = 4‐NO_2_Ph), **101b** (IC_50_ = 2.05 µM; R^1^ = H, R^2^ = 4‐BrPh), **101c** (IC_50_ = 4.12 µM; R^1^ = H, R^2^ = 2,4‐diClPh), and **101d** (IC_50_ = 1.72 µM; R^1^ = CH_3_, R^2^ = 4‐BrPh) stood out (Scheme 30). The ability of the compounds to inhibit the growth of promastigote and amastigote forms of *Leishmania amazonensis* and *Leishmania infantum* was also investigated. Compound **101d** showed an IC_50_ value of 6.17 µM against *L. amazonensis*, compared to the standard drug miltefosine, which presented an IC_50_ of 15.83 µM. For *L. infantum*, compound **101d** exhibited an IC_50_ value of 6.04 µM. Most compounds, however, displayed IC_50_ values above 200 µM. The pharmacokinetic evaluation conducted using SwissADME demonstrated that all synthesized compounds were compliant with Lipinski's rule. Additionally, the analysis of the topological polar surface area (TPSA) indicated that all compounds exhibited appropriate values. Most compounds showed high gastrointestinal absorption and good oral bioavailability, along with great chemical stability.

**SCHEME 31:**
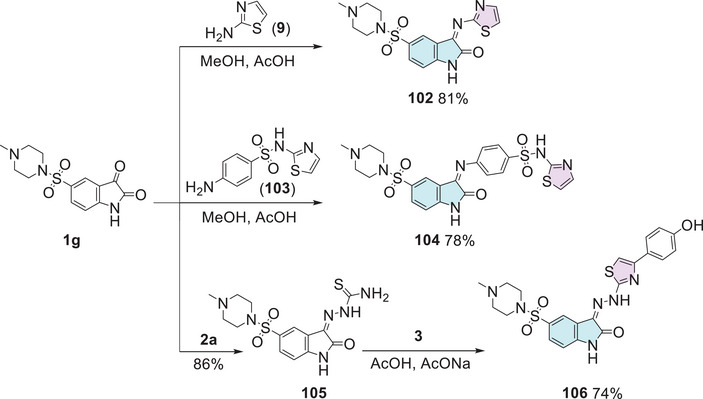
Synthetic route of compounds **102**, **104**, and **106**.

In the quest for potent antibacterial and antifungal agents, Alzahrani et al. [144] designed a series of novel hybrid heterocyclic agents based on isatin, thiazole, and sulfonamine in search of compounds with significant antibacterial and antifungal activity. The synthetic strategy to afford the hybrids was in three pathways. Here 5‐((4‐methylpiperazin‐1‐yl) sulfonyl) indoline‐2,3‐dione **1g** was used as a common intermediate to three different types of products. The first was using **1g** reacting with thiazol‐2‐amine **9** to promote a condensation at the C‐3 position to afford **102** (81% yield). The combination of **1g** and 4‐amino‐*N*‐(thiazol‐2‐yl)benzenesulfonamide **103** promotes Schiff's base product **104** (78% yield). The **1g** compound, when reacted with thiosemicarbazide **2a**, undergoes a condensation at the C‐3 position, forming **105**, which then reacts with alpha‐bromoacetophenone **3**, resulting in the desired product **106** with 74% yield (Scheme 31).

In vitro testing was conducted against three Gram‐positive bacterial strains, three Gram‐negative strains, and one fungal pathogen, using levofloxacin and nystatin as positive controls. The compound **104** exhibited the highest activity MIC values (7.8–15.6 µg/mL) against *S. aureus* (ATCC 25923), *S. aureus* (ATCC 29213), and *Enterococcus faecalis* (ATCC 29212) compared to levofloxacin (32.5, 16.2, and 8.1 µg/mL, respectively) (Table 9). This enhanced activity is attributed to the presence of both sulfonyl and thiazole groups, which contribute to its broad‐spectrum antibacterial effect. Furthermore, compound **106** (Scheme 31), a 3‐(2‐(4‐(4‐hydroxyphenyl)thiazol‐2‐yl)hydrazono)indolin‐2‐one derivative, exhibited remarkable antibacterial potency, particularly against Gram‐negative bacteria. It displayed MIC values of 1.9–15.6 µg/mL, demonstrating up to 35‐fold higher activity compared to the standard drug levofloxacin (MIC = 65–130 µg/mL) (Table 9). Notably, compound **106** was the most active against *E. coli* (ATCC 35218), with an MIC of 1.9 µg/mL, highlighting its strong potential as an antibacterial agent. Regarding antifungal activity, all hybrids demonstrated weak‐to‐moderate values (62.5–500 µg/mL). Compounds **102**, **104**, and **106** were tested against *S. aureus* and *P. aeruginosa* biofilms, with compound **104** being the most active against *S. aureus* (IC_50_ = 1.95 µg/mL), whereas **102** and **106** exhibited BIC_50_ values of 15.6 µg/mL. In the inhibition of the quorum sensing (QS) system in *E. faecalis*, compound **106** showed the highest inhibition of the system (83.9%), followed by **102** (81.49%) and **104** (71.12%). In DHFR inhibition in vitro, compound **106** was the most selective (IC_50_ = 40.71 nM), surpassing methotrexate, whereas **102** and **104** had submicromolar IC_50_ values (0.26 and 0.28 µM, respectively). Docking studies revealed that these compounds have lower energy gaps (Δ*E* = 2.91–3.06 eV) than levofloxacin (Δ*E* = 4.19 eV), suggesting greater ease of interaction with biological receptors. Compound **106** exhibited the lowest binding energy (−31.64 kcal/mol), demonstrating a strong interaction with DHFR. According to Lipinski's rule of five and Veber's rule, derivatives **102** and **106** were deemed drug‐like, whereas derivative **104** failed to meet certain criteria [144].

**TABLE 9:** Summary of the MIC values for compounds **102**, **104**, and **106**.

Gram‐positive strains—MIC (µg/mL)				
	Levofloxacin	102	104	106
*Staphylococcus aureus (ATCC 25923)*	32.5	31.2	7.8	15.6
*Staphylococcus aureus (ATCC 29213)*	16.2	62.5	7.8	15.6
*Enterococcus faecalis (ATCC 29212)*	8.1	62.5	7.8	7.8
*Bacillus subtilis (ATCC 6051)*	16.2	31.2	62.5	31.2

Gram‐negative strains—MIC (µg/mL)				
	Levofloxacin	102	104	106
*Escherichia coli (ATCC 35218)*	65	125	15.2	1.9
*Pseudomonas aeruginosa 1 (36ATCC 9027)*	130	125	62.5	15.6
*Pseudomonas aeruginosa (PAR.36)*	130	125	62.5	15.6
*Salmonella Typhimurium (ATCC 14028)*	65	62.5	62.5	7.8

Fungal—MIC (µg/mL)				
	Nystatin	102	104	106
*Candida albicans (ATCC 10213)*	3.9	62.5	500	125

**SCHEME 32:**
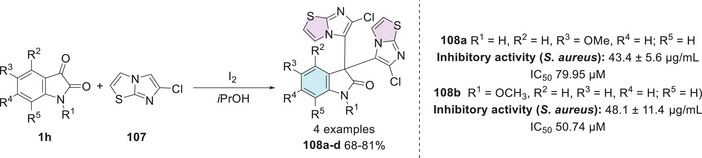
Synthetic method to achieve compounds **108a**–**d** and antibacterial activity for the best compounds.

Bonvicini et al. synthesized a series of new hybrids of isatin bis‐indole and isatin bis‐imidazothiazole, and their bioactivity was investigated against *S. aureus*, *E. coli*, and *C. albicans*. The synthesis of the four new compounds was carried out by reacting isatins **1h** with imidazo[2,1‐b]thiazole **107** in a condensation reaction to obtain a bis‐imidazothiazole at the C‐3 position **108a–d** with 68%–81% yields (Scheme 32). Regarding antibacterial activity, all compounds demonstrated activity against *E. coli* and *S. aureus*. Derivatives **108a** (43.4 ± 5.6 µg/mL, R^1^ = H, R^2^ = H, R^3^ = OMe, R^4^ = H; R^5^ = H) and **108b** (48.1 ± 11.4 µg/mL, R^1^ = H, R^2^ = H, R^3^ = H, R^4^ = H; R^5^ = H) (Scheme 32) were highly active against the Gram‐positive strain *S. aureus* but showed no inhibitory activity against *E. coli*. As for antifungal activity, none of the compounds significantly inhibited the growth of *C. albicans*. Due to their ability to inhibit *S. aureus* growth at non‐toxic concentrations, **108a** and **108b** were tested in dose‐response experiments to determine their IC_50_ values. Derivative **108a** exhibited an IC_50_ of 79.95 µM (39.12 µg/mL), whereas compound **108b** showed an IC_50_ of 50.74 µM (24.83 µg/mL). The isatin derivatives **108a** and **108b** demonstrated efficacy against 10 clinical isolates of *S. aureus* with different antibiotic susceptibilities, including both methicillin‐sensitive (MSSA) and methicillin‐resistant (MRSA) strains, with IC_50_ values similar to the reference strain, ranging from 73.01 to 85.31 µM (**108a**) and 45.02 to 59.38 µM (**108b**) [145].

**SCHEME 33:**
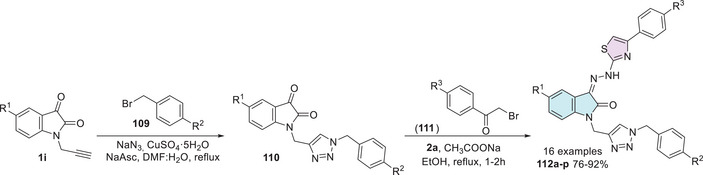
Synthetic route for compounds **112a**–**p**.

In prokaryotic organisms such as bacteria, DNA gyrase is an essential enzyme involved in the replication process, as it facilitates the duplication of genetic material. Inhibition of this enzyme not only halts bacterial proliferation but also induces cell death by causing DNA damage. Similarly, sterol 14α‐demethylase plays a critical role in fungal cell membrane biosynthesis, making it a valuable target for antifungal therapies. To enhance pharmacological potential, heterocyclic cores with known biological activity have been fused with isatin. In this context, Kumar et al. [146] designed a new class of 16 thiazole‐isatin‐1,2,3‐triazole hybrids and alkynyl isatin–thiazole hybrids and conducted an in silico evaluation of their biological potential using molecular docking and molecular dynamics calculations against DNA gyrase (1KZN; *E. coli*) and sterol 14α‐demethylase (5TZ1; *C. albicans*). The synthesis of the thiazole‐isatin‐1,2,3‐triazole hybrids **112a–p** was carried out in two steps. Initially, *N*‐propargylated isatin derivatives **1i** react with benzyl bromide derivatives **109**, sodium azide, CuSO_4_·5H_2_O, and sodium ascorbate in a Huisgen cycloaddition through alkylazide formation in situ pathway, which results in the formation of isatin–triazole hybrids **110**. Subsequently, these hybrids **110** react with phenacyl bromide derivatives **111** combined with **2a**, affording the desired isatin–triazole–thiazole hybrids **112a–p** in 76%–92% yields (Scheme 33). A new class of thiazole–isatin–1,2,3‐triazole hybrids **112a–p** was evaluated as to its biological potential through in silico studies. Molecular docking and molecular dynamics calculations were conducted against two key biological targets: DNA gyrase (1KZN) from *E. coli* and sterol 14α‐demethylase (5TZ1) from *C. albicans*. These enzymes play crucial roles in bacterial replication and fungal sterol biosynthesis, respectively, making them strategic targets for developing new antimicrobial agents. Sterol 14α‐demethylase is essential for fungal sterol biosynthesis, as these sterols contribute to the structural integrity of mitochondria, the endoplasmic reticulum, peroxisomes, and the plasma membrane. Consequently, inhibiting this enzyme disrupts multiple cellular processes vital for fungal survival, highlighting its significance as an antifungal target [147, 148, 149, 150]. Structure–activity relationship (SAR) analysis revealed that the presence of Br in the hybrid improves interactions with both enzymes; for instance, compound **112a** (R^1^ = Br, R^2^ = F, R^3^ = OMe) exhibited binding affinities with 1KZN and 5TZ1 of −10.3 and −12.6 kcal/mol, respectively (Scheme 33). Molecular dynamics simulations were performed for compound **112a**, calculating the root mean square deviation (RMSD) and root mean square fluctuations (RMSF). For the 1KZN‐53 complex, the RMSD value was 0.175 nm, with RMSF values ranging from 0.05 to 0.25 nm. In contrast, for the 5TZ1‐53 complex, the RMSD ranged from 0.15 to 0.25 nm, whereas the RMSF values varied between 0.05 and 0.35 nm. Therefore, these molecular hybrids show potential as effective scaffolds for further evaluation of their antimicrobial activity [146].

**SCHEME 34:**
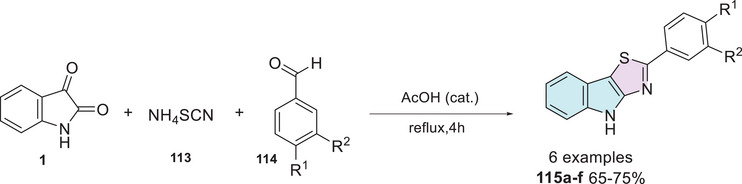
Synthetic route for compounds **115a**–**f**.

Posinasetty et al. [151] synthesized a library of 2‐aryl‐4*H*‐[1,3]‐thiazolo[4,5‐*b*]indoles to explore the SAR and optimize their pharmacological potential for antibacterial activity. The synthesis of the six hybrid compounds was carried out by mixing isatin **1** and ammonium thiocyanate **113** to form a C–S bond at C‐3 position, followed by condensation with an aryl aldehyde **114** to form thiazol cyclic compound at C‐2 and C‐3 positions of isatin derivative as the desired product **115a–f** (65%–75% yields, Scheme 34). The in silico study revealed that all derivatives comply with Lipinski's rule, exhibit high gastrointestinal absorption, good blood–brain barrier (BBB) permeability, and show no dermal penetration or structural alerts for pan‐assay interference compounds (PAINS), reinforcing their potential as drug candidates. Molecular docking studies using Glide showed that compounds **115a** (R^1^ = Br, R^2^ = H) and **115b** (R^1^ = H, R^2^ = NO_2_) exhibited the best interactions with the target proteins 2PR2 and 1S14, validated by RAMPAGE, ERRAT, and Verify 3D. Among the key biological targets investigated, topoisomerases play a crucial role in DNA preservation and replication across various organisms, ensuring normal cell function. Their inhibition disrupts DNA replication, leading to genetic damage and ultimately inducing cell death. This mechanism is widely exploited in the treatment of both microbial infections and cancer, making topoisomerase inhibitors highly valuable therapeutic agents [152]. Molecular docking analysis using Schrödinger software indicated that compounds **115a** and **115b** were the most active against *Mycobacterium tuberculosis* and *E. coli* topoisomerase. Antitubercular activity was assessed using isoniazid (INH) as the standard drug, and compounds **115c** (R^1^ = OH, R^2^ = H) and **115d** (R^1^ = OMe, R^2^ = H), containing *p*‐hydroxy and *p*‐methoxy groups, demonstrated activity at all tested concentrations of 25, 50, and 100 µg/mL. Regarding antibacterial activity, compounds **115a** and **115e** (R^1^ = Cl, R^2^ = H) showed the highest efficacy, attributed to the presence of *p*‐chloro and *p*‐bromo groups (Scheme 34).

**SCHEME 35:**
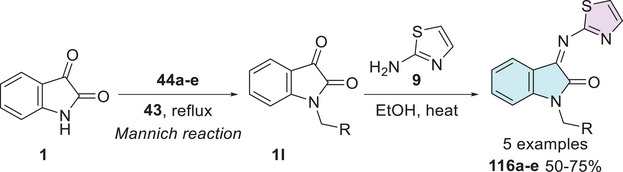
Synthetic method to afford the compounds **116a**–**e**.

Al‐Musawi and Al‐Mudhafar [153] conducted a theoretical study using molecular docking and ADMET analyses, alongside an experimental investigation of isatin–thiazole hybrids. The synthesis strategy initially involved isatin **1** and the preparation of Mannich's bases, using a series of secondary amines **44** (morpholine, piperidine, pyrrolidine, *N*‐methylaniline, diethylamine) and a formaldehyde **43**, to react to obtain the alkylated intermediate **1l**, which reacts with **9**, to afford the corresponding Schiff's base at C‐3 carbonyl **116a–e** in 50%–75% yields (Scheme 35).

The antimicrobial activity was evaluated against two Gram‐positive bacteria (*S. aureus* and *Bacillus licheniformis*), two Gram‐negative bacteria (*E. coli* and *Acinetobacter baumannii*), and the fungus *C. albicans* (Table 10). Regarding antifungal activity, only compounds **116a–d** exhibited moderate‐to‐strong inhibition zones against *C. albicans* (15–27 mm), compared to the standard drug fluconazole (20 mm). In terms of antibacterial activity, most compounds were active against all tested strains, showing promising inhibition zones (5–20 mm) relative to fluconazole (13–50 mm) [153].

**TABLE 10:** Zone of inhibition in mm of compounds **116a**–**d**.

Compounds	Gram‐positive		Gram‐negative		Fungi
	*Staphylococcus aureus*	*Bacillus licheniformis*	*Escherichia coli*	*Acinetobacter baumannii*	*Candida albicans*
**116a** (R = morpholine)	17	—	17	5	15
**116b** (R = piperidine)	15	15	17	16	21
**116c** (R = pyrrolidine)	15	10	17	12	20
**116d** (R = diethylamine)	20	17	18	15	27
**Ciprofloxacin**	35	50	40	13	—
**Fluconazole**	—	—	—	—	20

Molecular docking studies revealed that compounds **116d** (R = diethylamine) and **116b** (R = piperidine) had stronger binding affinities than fluconazole, particularly at the 1EA1 binding site. Among them, **116d** exhibited the highest binding score (−7.3 kcal/mol), followed by **116b** (−7.07 kcal/mol). Compared to ciprofloxacin, compounds **116c** (R = *N*‐methylaniline) and **116d** also showed superior performance, with binding scores of −6.535 and −5.488 kcal/mol, respectively, suggesting a higher inhibitory potential. Structural analysis indicated that the presence of indole, thiazole, and cyclic secondary amines in these compounds enhances interactions such as hydrogen bonding, π–cation interactions, and π–π stacking. Additionally, the compounds complied with Lipinski's rule of five and demonstrated good oral absorption. Notably, compound **116d** stood out for its lack of penetration into the CNS, suggesting a lower risk of CNS‐related side effects [153].

### 5.7 Antioxidant

Antioxidant activity is related to the inhibition or retardation of molecular oxidation in living organisms. Antioxidants play a crucial role in maintaining cellular integrity by protecting lipids, proteins, and DNA from free radical attacks, thereby preventing mutations and degenerative processes. Uncontrolled oxidation can contribute to premature aging and the development of various diseases, including cancer, cardiovascular diseases, and neurodegenerative disorders. In vitro and animal model studies have been widely used to explore new potential antioxidants, aiming to enhance cellular protection and minimize oxidative damage [154, 155].

**FIGURE 6:**
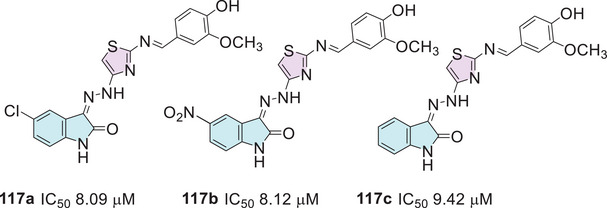
Antioxidant activity of **117a**–**c**.

In this context, Eggadi et al. [23] evaluated the in vitro antioxidant potential of 23 isatin‐3‐[*N* ^2^‐(2‐benzalamino‐thiazol‐4‐yl)] hydrazone conjugates. The synthetic route for obtaining the compounds followed the approach proposed in Scheme 29. Overall, all compounds exhibited mild to potent antioxidant activity. Compounds **117a**, **117b**, and **117c** demonstrated potent IC_50_ values of 8.09, 8.12, and 9.42 µM, respectively (Figure 6). The remaining compounds showed moderate‐to‐mild activity, with IC_50_ values ranging from 8.12 to 10.12 µM, compared to the reference compound ascorbic acid (IC_50_ 5.87 µM). The high antioxidant activity was attributed to the presence of donor groups, which reduce the DPPH free radical (2,2‐diphenyl‐1‐picrylhydrazyl) and help prevent cellular damage.

**SCHEME 36:**
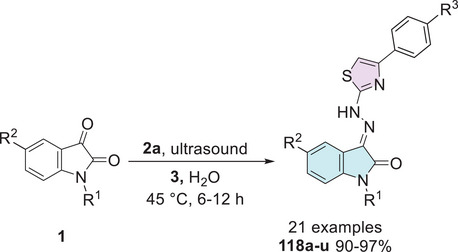
Synthesis of compounds **118a**–**u**.

Khanum and Pasha [155] developed, for the first time, a catalyst‐free synthesis of 21 isatin–thiazole hybrids (**118a**–**u**) with yields of 90%–97% in a single step using ultrasound. The reaction involved isatin derivatives (**1**), thiosemicarbazide (**2a**), and alpha‐bromoacetophenone derivatives (**3**), which react to form a Schiff base at the C‐3 carbonyl of isatin, incorporating a thiazole nucleus (Scheme 36).

Additionally, the antioxidant activity of the synthesized compounds was evaluated. Among the tested compounds, **118a** (R^1^ = H, R^2^ = F, R^3^ = F) exhibited a highly potent IC_50_ value of 5.02 µg/mL, outperforming the standard drug ascorbic acid (IC_50_ 9.01 µg/mL) (Table 11). The remaining compounds demonstrated moderate antioxidant activity, with IC_50_ values ranging from 11.76 to 35 µg/mL. Compounds with N‐alkyl groups (IC_50_ of 11.76–78.24 µg/mL) showed superior activity compared to those with N‐benzyl groups (IC_50_ 303 µg/mL) (Table 11). Furthermore, the presence of fluorine positively influenced activity, showing greater effectiveness than iodine and chlorine. When comparing 4‐cyano, 4‐fluoro, and 4‐methoxyphenacyl bromides, antioxidant activity followed the decreasing order: F > OCH_3_ > CN [155].

**TABLE 11:** The IC_50_ values of active compounds **118a**–**d**.

Compounds	IC_50_ (µM)
**118a** (R^1^ = H, R^2^ = F, R^3^ = F)	5.02
**118b** (R^1^ = CH_3_, R^2^ = H, R^3^ = F)	18.09
**118c** (R^1^ = C_2_H_5_, R^2^ = H, R^3^ = F)	32.02
**118d** (R^1^ = Bn, R^2^ = H, R^3^ = F)	303
**Ascorbic acid**	9.01

A study using SwissADME prediction software identified compound **118a** as the most promising candidate, suitable for both oral administration and intravenous therapy. Analysis of pharmacokinetic properties revealed that most compounds exhibited high TPSA values. Skin permeability, a crucial factor for transdermal absorption, was also assessed; all tested compounds showed negative log *K*_p_ values (−6.24 to −0.96), within the acceptable range of −8.0 to −1.0, indicating good skin permeability. However, most compounds did not demonstrate significant ability to cross the BBB, although compounds **118b** (R^1^ = CH_3_, R^2^ = H, R^3^ = F), **118c** (R^1^ = C_2_H_5_, R^2^ = H, R^3^ = F), and **118d** (R^1^ = Bn, R^2^ = H, R^3^ = F) (Scheme 36) did show BBB permeability. Only compound **118a** effectively inhibited *P*‐glycoprotein (*P*‐gp), a crucial factor for efficient drug delivery to the CNS [155].

### 5.8 Cytotoxic Activity

**SCHEME 37:**
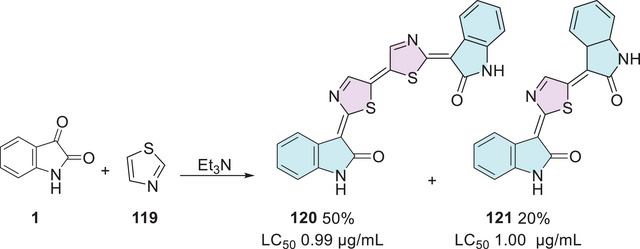
Synthesis and cytotoxic activities of compounds **120** and **121**.

Hoque and Islam [156] investigated the cytotoxic activity of indophenines using *Artemia salina* nauplii as a model. The synthesis was achieved by mixing free isatin (**1**) and thiazole (**119**) in triethylamine, promoting either a mono‐insertion (**120**) or bis‐insertion (**121**) of the thiazole ring linked between two isatin units, resulting in yields of 50% and 20%, respectively, as shown in Scheme 37. Both conjugates exhibited high cytotoxic activity, with LC_50_ values of 0.99 and 1.00, respectively. SAR analysis correlated the significant bioactivity of these compounds to the presence of nitrogen (N) and sulfur (S) atoms in the thiazole ring.

**SCHEME 38:**
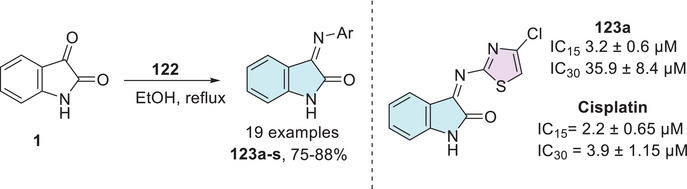
Synthetic route for compounds **123a**–**s**, highlighting activity for the best compound.

Azizian et al. [157] conducted cytotoxicity assays and molecular docking studies on several Schiff bases derived from isatin. The synthesis of nineteen compounds (**123a**–**s**) was carried out using free isatin (**1**) and primary amines (**122**) to afford the desired Schiff bases, with yields ranging from 75% to 88% (Scheme 38). Most of these bases exhibited high cytotoxic activity in HeLa cells. Compounds containing electron‐withdrawing groups in their scaffold showed the best results, particularly compound **123a** (Ar = 4‐chlorothiazol‐2‐yl, Scheme 38), which features a five‐membered chlorothiazole heterocyclic ring with an electronegative chlorine atom at the fourth position (HeLa cells—IC_15_ = 3.2 ± 0.6 µM; IC_30_ = 35.9 ± 8.4 µM) when compared to the standard drug, which showed IC_15_ = 2.2 ± 0.65 µM and IC_30_ = 3.9 ± 1.15 µM. The docking study was performed by redocking the co‐crystallized conformation of cognate ligands into the three‐dimensional structure of VEGFR‐2, highlighting the importance of the NH group of indoline, which facilitates cisplatin hydrogen bonding between the carbamate NH and Cys917. Furthermore, the results indicated that the presence of extended hydrophobic rings and hydrogen bond acceptor groups at the third position of the indoline scaffold could contribute to the development of potent VEGFR‐2 inhibitors.

**FIGURE 7:**
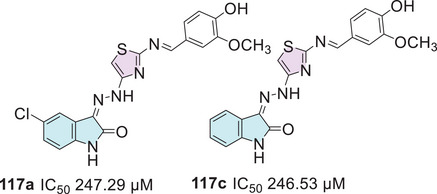
Cytotoxic activity against the HBL‐100 and HeLa cell lines of **117a** and **117c**.

Eggadi et al. [23] used cisplatin (IC_50_ = 25 µM) as a standard to evaluate the cytotoxicity of isatin conjugates against the HBL‐100 and HeLa cell lines. Among the 10 compounds analyzed, only compounds **117a** and **117c** (Figure 7) exhibited cytotoxic activity, with IC_50_ values of 247.29 and 246.53 µM, respectively. The synthetic route was presented in Scheme 29. The presence of a halogen atom at Position 5 appeared to influence biological activity, as electronegative atoms increase the lipophilicity of molecules, a factor associated with enhanced cytotoxicity in the MTT model.

## 6 Conclusion and Outlook

This review gives an elaborate knowledge of the synthesis of isatin–thiazole hybrids by different methodologies, which play a significant role as multifunctional bioactive compounds, supported by a broad spectrum of pharmacological activities. Their structural versatility, combined with the reactivity of the isatin core and the biological relevance of the thiazole ring, underscores their value as a promising scaffold for drug development. The growing number of studies published up to 2024 reflects the continued interest of the scientific community in exploring these hybrids for therapeutic applications, particularly in tackling challenges related to cancer, infectious diseases, inflammation, and metabolic disorders. From a synthetic point of view, the major challenge is mainly focused on the anchoring points of the isatin nuclei; that is, the vast majority of examples involve the coupling of the thiazole nucleus to the isatin B ring. In the coming years, research is expected to focus on optimizing the pharmacokinetic and pharmacodynamic properties of isatin–thiazole derivatives through SAR studies, targeted molecular modifications, and in vivo evaluations. Additionally, advancements in computational modeling, sustainable synthetic methodologies, and high‐throughput screening may accelerate the identification of promising lead candidates. Overall, isatin–thiazole hybrids remain a fertile platform for innovation, offering promising opportunities for the development of safer and more effective therapeutic agents.

## Conflicts of Interest

The authors declare no conflicts of interest.

## Data Availability

The authors have nothing to report.
